# Full-Length Transcriptome Survey and Expression Analysis of Parasitoid Wasp *Chouioia cunea* upon Exposure to 1-Dodecene

**DOI:** 10.1038/s41598-019-54710-0

**Published:** 2019-12-03

**Authors:** Lina Pan, Meiqi Guo, Xin Jin, Zeyang Sun, Hao Jiang, Jiayi Han, Yonghui Wang, Chuncai Yan, Min Li

**Affiliations:** 1 Tianjin Key Laboratory of Animal and Plant Resistance, Tianjin Normal University, Tianjin, 300387 China; 2 South China University of Technology, 381 Tianhe Road, Guangzhou, 510641 China

**Keywords:** Next-generation sequencing, Entomology

## Abstract

*Chouioia cunea* (Yang) is an endoparasitic wasp which parasitizes pupae and thus plays an important role in the biological control of the fall webworm (*Hyphantria cunea* Drury), an important quarantine pest in the entire world and a major invasive pest in China. For the purposes of investigating which proteins are involved in the response of *C. cunea* to 1-Docecene, one of the chemical compounds of pupae of *H. cunea* with a significant attracting action to mated female *C. cunea*, 11.5 Gb transcriptome data was sequenced on the PacBio RS II platform from 1-day old *C. cunea* adults to generate a reference assembly. Afterwards, 46.88 Gb of clean RNA-Seq data were obtained to assess the transcriptional response of these insects before and after the stimulation with 1-Docecene. After removing redundancy using CD-HIT, a sequence structure analysis predicted 29,105 complete coding sequence (CDS) regions, 51,458 single-sequence repeats (SSRs), and 2,375 long non-coding RNAs. Based on the early transcriptome sequencing in our laboratory, we revealed some new sequences corresponding to chemosensory genes such as odorant binding proteins (OBPs), odorant receptor (OR), gustatory receptors(GRs). Results of quantitative real-time PCR experiments revealed that CcOBP7, CcOBP18, CcCSP4, CcOR2, and CcGR18 were up-regulated after 1-Dodecene stimulation. In addition, the expression of 31 genes, including 1 gene related to phospholipid biosynthesis and 2 genes related to transmembrane transport were up-regulated after 1-Dodecene stimulation; meanwhile, the expression of 22 genes, including 5 genes related to protein phosphorylation and protein serine/threonine kinase activity were significantly down-regulated after 1-Dodecene stimulation. These results suggest that the attraction of adult *C. cunea* to 1-dodecane is associated with the transmembrane signal transduction and dephosphorylation of some proteins. Our findings will provide useful targets for further studies on the molecular mechanism of host recognition in *C. cunea*.

## Introduction

In insects, the olfactory system is mainly used to communicate with the outside environment, forage, evade enemies, conduct courtship and mating, locate hosts, and select mating sites^1^. The complex process of olfactory recognition involves various protein molecules, including odorant binding proteins (OBPs), chemosensory proteins (CSPs), odorant receptors (ORs), gustatory receptors (GRs),and sensory neuron membrane proteins (SNMPs)^2–5^. The majority of existing researches based on insect olfaction have mainly focused on some harmful species. Therefore, studies on natural enemies, particularly parasitic insects, are lacking despite the important role of species such as *Chouioia cunea* Yang in biological pest control^6^.

The fall webworm, *Hyphantria cunea* (Drury) (Lepidoptera: Arctiidae), is an invasive and quarantined pest^7^. It is a highly polyphagous insect which has the ability to attack a variety of plants, including a wide range of tree species and agricultural crops, especially broad-leaf trees. The life cycle of fall webworm consists of four different periods; egg, larva, pupa and adult, among which the larval stage of the pest is a process during which a large amount of leaves could be eaten, causing certain damage to forestry and agriculture. In China, *Chouioia cunea* Yang (Hymenoptera: Eulophidae) is currently considered the optimal insect species with the highest parasitism rate (exceeding 92.2%) against *H. cunea*, and can be used to achieve sustained, long-term control^8^. Moreover, *C. cunea* targets six different common alternate hosts, all of which are forest pests, including *Stilpnotia salicis, Ivela ochropoda, Clostera anachoreta, Semiothisa cinerearia, Clania variegeta, Acronycta intermelia*^9^. The reproductivity of parasitoid wasps depends on the ability to locate the hosts^10^ and infochemicals provide cues for host location^11^. Despite its importance, the molecular mechanism via which *C. cunea* recognizes its hosts remains poorly understood because of a shortage of genomic data and lack of information regarding the volatiles that attract these wasps. Although we have previously demonstrated that 1-Dodecene, one of the chemical compounds of pupae of *H. cunea*, could elicit a significant EAG response and a attracting action to mated female *C. cunea*^12^, the molecular mechanism underlying this olfactory response remains unclear. However, the chemosensory genes are the ideal targets for understanding the olfactory code of insects.

Transcriptome research is indispensable to our understanding of biological processes. We previously used next-generation transcriptome sequencing by a 454 GS-FLX sequencer to identify some chemosensory genes in *C. cunea*, including 25 CcOBPs, 11 CcCSPs, 1 CcOrco, 79 CcORs, 17 GRs, 10 IRs, and 1 SNMP^13^. However, all the next-generation sequencing technologies based on transcriptome assemblies generated with short-reads were generated have shortage to yield complete, accurately assembled transcripts or to recognize transcripts expressed in terms of isoforms, homologous genes, superfamily genes, and alleles. These limitations present challenges to a deeper understanding of biological mechanisms. In contrast, full-length transcriptome sequencing based on PacBio SMRT single-molecule real-time (SMRT) sequencing technology is powered by the long-read sequencing platform. This ultra-long reading capacity (median: 10 kb) thus provides data corresponding to single complete transcript sequences. Accordingly, the post-analysis process requires no assembly, and the measured data can be used directly^14,15^. However, to yield a final set of non-redundant transcript sequences, CD-HIT^16^ software was used to merge highly similar sequences and remove redundant sequences from high-quality transcript.

In this study, to obtain the full-length transcriptome of *C. cunea* and to detect which proteins are involved in the response to 1-Docecene, full-length transcriptome sequencing approach based on the PacBio RS II platform, in combined with the next-generation sequencing technology based on Illumina HiSeq platform were used. Genes expressed differentially in insects before and after 1-Dodecene stimulation were screened, and the NR^17^, Swissprot^18^, Gene Ontology (GO)^19^, Cluster of Orthologous Groups of proteins (COG)^20^, Eukaryotic Ortholog Groups (KOG)^21^, Pfam^22^ and KEGG^23^ databases were used to obtain the annotations. Given this approach, our study may lay a foundation for elucidating the molecular mechanism underlying the olfactory response of *C. cunea* to 1-Dodecene.We hope that this study of the olfactory mechanism employed by parasitic wasps will improve our understanding of the intra-specific or inter-specific chemical communication used by parasitic insects and thus provide a theoretical basis for regulating biological control via corresponding chemical signals.

## Results

### PacBio sequencing and error correction of long reads

A cDNA library was prepared using RNA isolated from 1-day-old *C. cunea* adults sequenced using SMRT technology and the Pacbio RS high-throughput sequencing platform. A total of 15,041 polymerase reads were obtained. After filtering low-quality data (sequences with polymerase read fragment lengths <50 bp and a predicted consensus accuracy <0.80), the adapter and primer sequences in the reads were truncated and 11.15 Gb of clean data were obtained. These raw data yielded 609,706 ROIs (reads of inserts) based on full passes ≥0 and a predicted consensus accuracy ≥0.8. The mean insert read length, read quality, and number of passes were 2,457 bp, 0.91, and 6, respectively (Table 1).

**Table 1:** PacBio libraries and sequencing results.

cDNA size	Reads of Insert	Read Bases of Insert	Mean Read Length of Insert	Mean Read Quality of Insert	Mean Number of Passes
1–6 K	609,706	1,498,604,382	2,457	0.91	6
All	609,706	1,498,604,382	2,457	0.91	6

**Figure 1:**
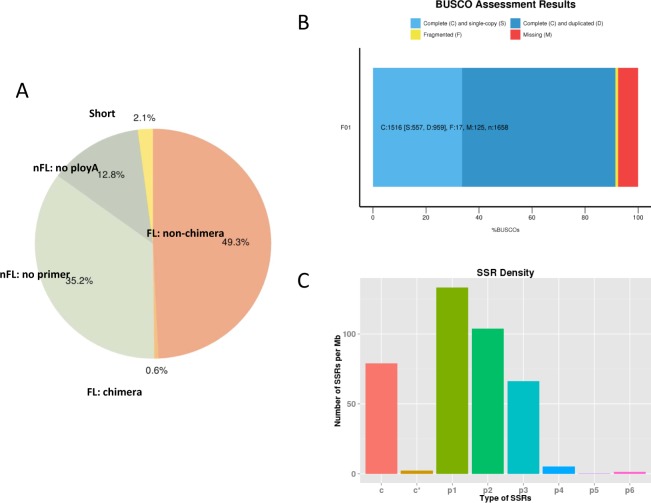
PacBio sequencing and SSR detection. (**A**) ROIs (reads of insert) classification. (**B**) BUSCO assessment results. (**C**) SSR size distribution. X axis represents the type of SSR. Y axis represents the number of SSR.

All ROIs were further classified into FL, non-FL (nFL), and FLNC transcripts as described in the Methods. We obtained 300,304 FL non-chimeric reads (49.3%) with a mean length of 2,485 bp, 214,617 no primer (35.2%), 78,042 no poly-A(12.8%), 12,647 filtered short reads (2.1%), as well as 3,530 FL chimeric reads (0.6%) (Fig. 1A, Table 2). The lengths of the FL sequences reflected the lengths of the cDNA sequences used to construct the library and could also be used to determine the quality of the library. These data yielded FL sequence lengths consistent with the size of the library (Fig. S1). The proportion of artificial concatemers was 1.16%, suggesting that a moderate SMRTbell concentration and credible data.

**Table 2:** Summary of sequencing reads after filtering.

cDNA Size	Reads of Insert	Number of filtered short reads	Number of nFL reads	Number of FL reads	Number of FL non-chimeric reads	Number of FL chimeric reads	Average FL non-chimeric read length
1–6 K	609,706	12,647	293,225	303,834	300,304	3530	2,485
All	609,706	12,647	293,225	303,834	300,304	3530	2,485

FL: full-length

nFL: non-full-length.

Furthermore, iterative sequence clustering was performed using the ICE algorithm with the SMRT Analysis software. Finally, 151,846 consensus isoforms were obtained, with a mean read length of 2,754 bp. The Quiver program was used to correct the consistent sequences in each cluster together with non-FL sequences, yielding 26,296 high-quality transcripts (high-quality isoforms) with accuracies exceeding 99%. Furthermore, to improve the accuracy of the isoforms, the low-quality transcripts were corrected with the corresponding Illumina sequencing data by proovread software. Finally, 36,560 non-redundant transcript sequences were obtained after using CD-HIT^16^ software as described in the Methods.

The integrity of the dereplicated transcriptomes was evaluated using Benchmarking Universal Single-Copy Orthologs (BUSCO)^24^, version 3.0.2 (Fig. 1B). A total of 1,658 BUSCO groups were searched, including 1516 complete BUSCOs [C,91.4%; including 557 single-copy (S) and 959 duplicated BUSCOs (D)], 17 fragmented BUSCOs (F,1.0%), and 125 missing BUSCOs (M,7.6%) (Fig. 1B), suggesting that the integrity of the dereplicated transcriptomes is reliable.

### Alternative splicing and SSR detection

All non-redundant transcript sequences were aligned using BLAST^25^, and alternative splicing events were predicted. BLAST alignments could be considered as products of candidate AS events as long as they meet the following criteria: (1) both of the two alignments should be greater than 1000 bp, and from which there should be two HSPs (High-scoring Segment Pair); (2) two HSPs have the same forward/reverse direction within the same alignment, one sequence should be continuous, or with a small “Overlap” size(smaller than 5 bp); the other one should be distinct to show an “AS Gap”, the continuous sequence should pretty much completely align to the distinct sequence. The AS Gap should be larger than 100 bp and at least 100 bp away from the 3′/5′ end. As shown in Tables S1, 4,038 alternative splicing events were detected.

As shown in Table 3, 36,179 sequences longer than 500 bp were screened. A MISA software based SSR analysis revealed that 20,102 of these sequences contained a SSR and 11,849 contained more than 1 SSR. A total of 51,458 SSRs were identified, including 20,671 mononucleotides, 18,346 dinucleotides, 11,264 trinucleotides, 889 tetranucleotides, 46 pentanucleotides and 242 hexanucleotides (Table 3). The statistical analysis of the density distributions of different SSRs is shown in Fig. 1C.

**Table 3:** Summary of SSR.

Searching item	Numbers
Total number of sequences examined	36179
Total size of examined sequences (bp)	103356841
Total number of identified SSRs	51458
Number of SSR containing sequences	20102
Number of sequences containing more than 1 SSR	11849
Number of SSRs present in compound formation	10995
Mono nucleotide	20,671
Di nucleotide	18,346
Tri nucleotide	11,264
Tetra nucleotide	889
Penta nucleotide	46
Hexa nucleotide	242

### CDS detection and lncRNA prediction

**Figure 2:**
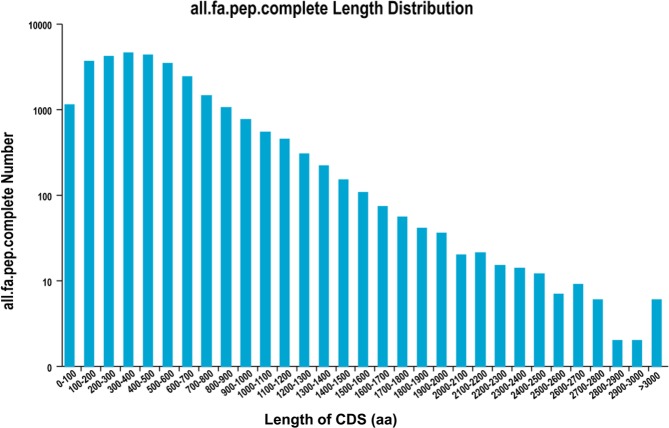
CDS length distribution. X axis represents the length of CDS. Y axis represents the number of CDS.

**Figure 3:**
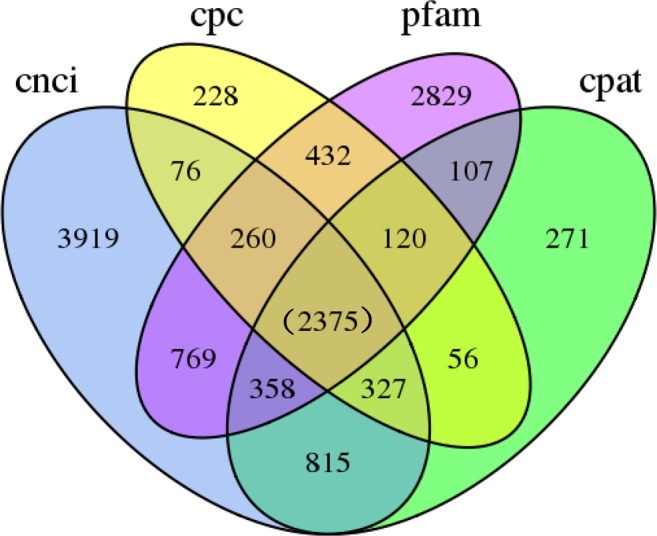
Venn diagram showing the overlap between CPC, CNCI, CPAT and Pfam.The circles represent the number of non-protein coding RNA candidates sorting by different computational approaches, and the number of non-coding RNAs identified by all 4 analysis methods was shown in brackets.

TransDecoder (https://github.com/TransDecoder/TransDecoder/releases) was used to identify the candidate CDS regions within transcript sequences. A total of 34,146 open reading frames (ORFs) were generated, of which 29,105 were complete. The length distribution of the complete ORF coding protein sequence is shown in Fig. 2. The combined computational approach based on CPC^26^/CNCI/CPAT^27^/Pfam was applied to sort non-protein-coding from putative protein-coding RNAs in the transcripts, as described in the Methods. A CPC analysis identified 3,874 non-coding RNAs (score <0) with a mean length of 2,068 bp. A CNCI analysis identified 8,989 non-coding RNAs (score <0) with a mean length of 2,128 bp. A CPAT analysis yielded 4,429 non-coding RNA with a mean length of 1,902 bp and mean ORF size of 240 bp. Furthermore, 7,250 non-coding RNA were obtained from the Pfam database. Subsequently, a Venn diagram was drawn to identify the intersecting non-coding transcripts identified through the above 4 analyses (Fig. 3). Briefly, 2,375 non-coding RNAs were identified by all 4 analysis methods. These transcripts were mainly candidate lncRNAs. Next, the LncTar target gene prediction tool was used to predict the target genes of these lncRNAs with a result of 857 genes, as shown in Table S5, Furthermore, an animal transcription factor database, animal TFDB 2.0^28^, was then used to predict a total of 772 transcription factors. As shown in Fig. S2, most predicted transcription factors were classified as miscellaneous (n = 592), followed by basic region-leucine zippers (TF_bZIP, n = 92), while more than 10 each were classified as NF-YC (Nuclear Factor Y, subunit C), NF-YB (Nuclear Factor Y, subunit B), and AP-2 (Activator protein-2) transcription factors.

### Annotation

**Figure 4:**
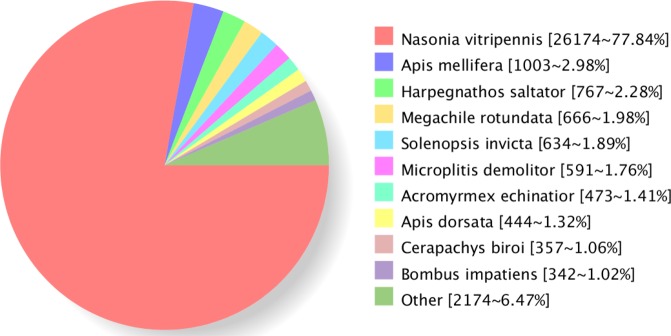
Distribution of NR annotated species. All non-redundant transcripts of *C. cunea* were used in tBLASTx to search the GenBank entries. The best hits with an E-value = 1.0E-5 for each query were grouped according to species.

TheBLAST^8^ alignment of the non-redundant transcript sequences with the Nr^29^, Swissprot^18,30^, GO^19^, COG^20^, KOG^21^, Pfam^22^, and KEGG^23^ databases yielded annotations for33,729 transcripts. The numbers of transcripts annotated in each database are shown in Table 4. Our blast alignment analysis revealed that the majority of our transcripts had highest scoring matches to proteins derived from *Nasonia vitripennis* (Hymenoptera: Pteromalidae) which is another parasitic wasp. Meanwhile, the total matching percentage of proteins derived from other species together besides *N. vitripennis* only presented a less than 30% similarity, further proving a close relationship of *N. vitripennis* and *C. cunea* (Fig. 4).

**Table 4:** Summary of functional annotation result.

Annotated databases	Isoform Number
NR	33,626
Swiss-Prot	23,218
GO	18,566
COG	13,154
KOG	25,947
Pfam	28,706
KEGG	17,669
eggNOG	32,586
All	33,729

Gene Ontology (GO) enrichment analysis was implemented through the tBLASTx program with an E-value less than 1.0e-5 to annotate 18,566 transcripts into 53 subcategories under the main functional groups of Biological Processes (12,729 transcripts), Molecular Function (15,741 transcripts), and Cellular Components (7,151 transcripts). Of the 19 subcategories in the Biological Process category, cellular process (9,116 transcripts, 23.3%), and metabolic process (9,845 transcripts, 25.2%) were the most well represented. Of the 18 subcategoriesin the Cellular Component category, the cell (5,280 transcripts, 20.9%) and cell part (5,284 transcripts, 20.9%) were most abundant. Of the 16 subcategories in the Molecular Function category, catalytic activity (8,611 transcripts, 38.2%), and binding (9,887 transcripts, 43.9%) were most represented (Fig. S2). However, 18,604 of the total 33,729 transcripts could be classified into 25 different functional COG categories (Fig. S3). Here, general function prediction only (4,687 transcripts, 25.19%), signal transduction mechanisms (1,517 transcripts, 8.15%), and transcription (1,572 transcripts, 8.45%) were the top 3 categories.

The Evolutionary genealogy of genes: Non-supervised Orthologous Groups (EggNOG) database was used to describe and classify groups of genes with homologous functions or something to that effect, leading to the annotation of 32,586 isoforms into 25 functional categories (Fig. S4). Here, function unknown (15,758 transcripts, 47.26%), post-translational modification, protein turnover, chaperones (2,916 transcripts, 8.74%), and signal transduction mechanisms (2,079 transcripts, 6.23%) were the top 3categoriesin this analysis. The KOG database was then used to annotate 25,947 isoforms into broad functional categories. The transcripts were classified into 25 KOG functional categories (Fig. S5), of which general function prediction only (4,159 transcripts, 16.03%), signal transduction mechanisms (2,755 transcripts, 10.62%), and post-translational modification, protein turnover, chaperones (2,159 transcripts, 8.32%) were the most well represented.

### Identification of chemosensory genes in *C. cunea*

As mentioned previously^13^, we performed tBLASTx analysis using available chemosensory protein sequences from hymenopteran species as “queries” to identify candidate chemosensory genes in the antennae of *C. cunea* in our earlier work, and all the genes mentioned were named 1, 2, 3, etc. according to the FPKM value. Based on that work, we identified 9 new OBPs, for which7 had corresponding FL CDSs. The nucleotide sequences of these putative transcripts are listed in Table S2. In this study, the FLs were supplemented to 4 partial CDSs of CSPs (CSP1, CSP2, CSP4, and CSP7)from the previous transcriptome assembly, and a new FLIR (IR75p) was discovered. Three new ORs were found, all of which were FL CDSs, and the FLs were supplemented to 5 original partial CDSs (OR22, OR48, OR53, OR68, and OR73). Moreover, 14 new GRs were identified, and all were FL CDSs. The original partial CDS GR6 was supplemented to a FL CDS.

### Differentially expressed genes (DEGs)associated with 1-Dodecene

**Figure 5:**
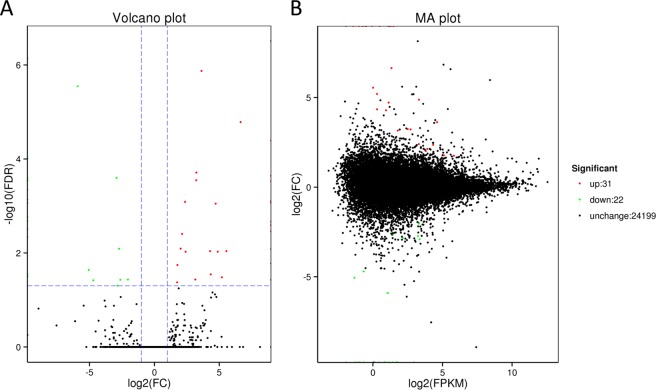
MA plot and Volcano plot of Differentially Expressed Genes (DEGs). (**A**) Volcano plot of DEGs. X axis represents -log10 transformed significance. Y axis represents log2 transformed fold change. Red points represent up regulated DEG. Blue points represent down regulated DEG. Black points represent non-DEGs. (**B**) MA plot of DEGs. X axis represents value A(log2 transformed mean expression level). Y axis represents value M(log2 transformed fold change). Red points represent up regulated DEG. Blue points represent down regulated DEG. Black points represent non-DEGs.

To investigate which proteins are involved in the *C. cunea* responded to 1-docecene, one of the chemical compounds of pupae of *H. cunea* with a significant EAG response and a attracting action to mated female *C. cunea*, the next-generation transcriptome sequencing was performed by Illumina platform with 6 samples, representing the control group and 1-Dodecene treatment group with 3 replicates per group, yielded 46.68 Gb of clean data. To avoid false-positives, the Benjamini–Hochberg correction method was used to correct the p-values obtained using the original test for significance. As shown in Fig. 5, 1-Dodecene treatment resulted in the up-regulation of 31 genes and down-regulation of 22 genes with a fold change ≥2 and false discovery rate (FDR) <0.01. These 53 DEGs were then subjected to a functional annotation enrichment analysis (Table S6). There were 34 genes annotated by the GO database, among which 17 genes (7 up-regulated and10 down-regulated) were classified into the Biological Process, 8 genes (5 up-regulated and 3 down-regulated) into Cellular Component, and 30 genes (16 up-regulated and 14 down-regulated) into Molecular Function categories, respectively. Five genes related to protein phosphorylation and protein serine/threonine kinase activity were significantly down-regulated, while 1 gene related to the phospholipid biosynthetic process and 2 genes related to transmembrane transport were significantly up-regulated.

Forty-two genes were annotated by the KOG database, including 24 up-regulated and 18 down-regulated genes. Five genes related to signal transduction (serine/threonine phosphorylation-related protein kinase) were down-regulated, while six genes related to intracellular substance transport were up-regulated. Possibly, *C. cunea* adults may be attracted to 1-dodecane via a mechanism involving transmembrane signal transduction and protein dephosphorylation.

### Chemosensory DEGs in response to1-Dodecene treatment

**Figure 6:**
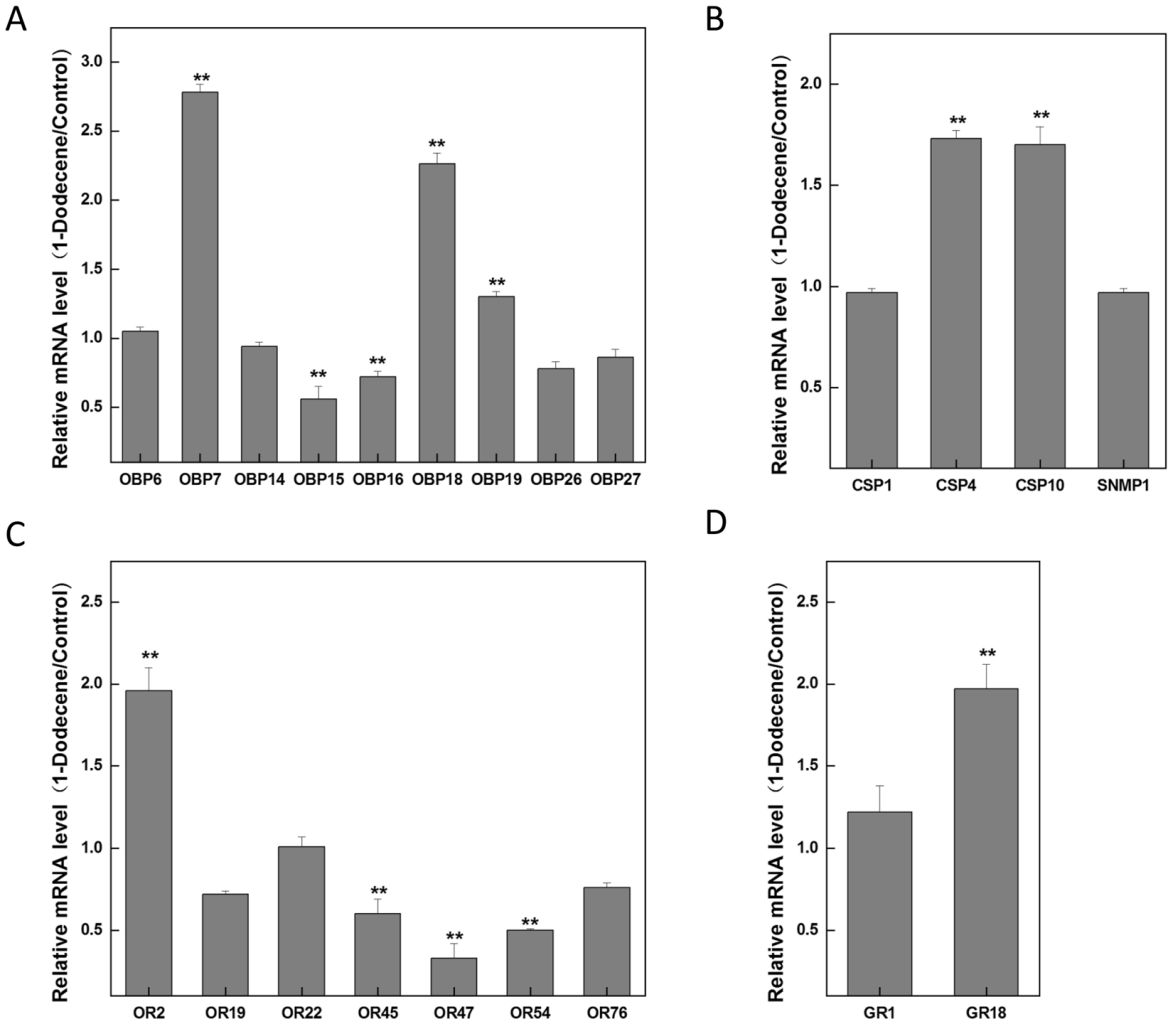
Relative expression levels of chemosensory genes in 1-Dodecene treatment measured by RT-qPCR. (**A**) Relative expression levels of OBPs in 1-Dodecene treatment. (**B**) Relative expression levels of CSPs and SNMP1 in 1-Dodecene treatment. (**C**) Relative expression levels of ORs in 1-Dodecene treatment. (**D**) Relative expression levels of GRs in 1-Dodecene treatment. The GAPDH was used to normalize transcript levels in each sample. The standard error is represented by the error bar (*p < 0.05, **p < 0.01).

To compare the accurate quantitative expression levels of chemosensory genes in response to 1-Dodecene, Real-time quantitative PCR (RT-qPCR) analysis was performed. Finally, 9 CcOBPs, 3 CcCSPs, 7 CcORs, 2 CcGRs, and 1 CcSNMP were subjected to RT-qPCR to determine the effects of 1-Dodecene treatment on the expression of chemosensory genes in *C. cunea* adults. These genes were selected according to a quantitative analysis of FPKM transcripts and differential expression among samples, using a fold change ≥2 as the screening criterion regardless of the p-value correction results. Among the 7selected CcOBPs, the expression levels of CcOBP7 and CcOBP18 after 1-Dodecene treatment were 2.78 and 2.26 times higher than that of the control (Fig. 6A), respectively. Pearson correlation coefficient analyses showed that there was a positive correlation between qPCR results and FPKM values (CcOBP7: Pearson correlation = 0.683, CcOBP18: Pearson correlation = 0.664). Moreover, the expression of CcOR2 after 1-Dodecene treatment was approximately twice that of the control, and there was a significant positive correlation between qPCR results and FPKM values (Pearson correlation = 0.979) (Fig. 6C). Therefore, we speculate that the direct binding of CcOBP7, CcOBP18, and 1-Dodecene, which are transported to olfactory receptor cells, may mediate the olfactory response of *C. cunea* adults to 1-Dodecene by activating the odor receptor CcOR2 and triggering nerve conduction. Moreover, the expression levels of CcCSP4 and CcCSP10 after 1-Dodecene treatment were 1.73 and 1.7 times higher than that of the control, respectively. There have been some reports stating that CSPs, widely expressed in different tissues and developmental stages of insects, have potential functions as carriers^31,32^. It can be inferred that CcCSP4 and CcCSP10 may act as the carrier for 1-Dodecene. In contrast, no significant difference was observed in the expression of CcSNMP from before to after treatment (Fig. 6B). However, the expression of CcGR18 after 1-Dodecene treatment was 1.97 times higher than that of the control (Fig. 6D). Pearson correlation coefficient analyses showed that there was a positive correlation between qPCR results and FPKM values (Pearson correlation = 0.719). Aside from the function in the detection of bitter compounds, sugars and non-volatile pheromones, thermotaxis, GRs were also reported to play a dual role in feeding and selecting an oviposition site^33–35^. We speculate that the CcGR18 may be involved in the response to 1-Dodecene of *C. cunea*. Taken together, these results suggest that CcCSP4, CcCSP10 and CcGR18 may also participate in the olfactory response of *C. cunea* adults to 1-Dodecene.

## Discussion

In our earlier study, a total of 25 OBPs, 80ORs, 10 IRs, 11 CSP, 1 SNMPs, and 17 GRs were annotated from antennal transcriptomes of *C. cunea*, but many of them had no full intact ORFs encompassing start and stop codons. One of the most important reasons why there was not extended with the long-read sequencing approach is the limitation that the next-generation sequencing technologies had shortage to yield complete, accurately assembled transcripts or to recognize transcripts expressed in terms of isoforms, homologous genes, superfamily genes, and alleles. Therefore, based on PacBio SMRT single-molecule real-time (SMRT) sequencing technology, we obtained more chemosensory genes with full length ORFs. However, it is unlikely that the identified genes represent the total number of the related chemosensory genes in *C. cunea*, because that transcripts presented in low abundance and those were too divergent to be identified using a BLAST search may have been overlooked in transcriptome analysis^36^.

Advances in chemical and behavioral ecology have directed attention toward the mechanisms governing the interactions among plants, phytophagous insects and their natural enemies^37^. Infochemicals could serve as bridges and links in these interactions. Chemical signals produced by hosts or host habitats play key roles in host seeking and the location of parasitic natural enemies. One such infochemical, 1-Dodecene, is associated with certain host stages (pupae) and clearly attracts the mated female adult *C. cunea*^12^. Here, we assessed changes in gene expression in *C. cunea* from before to after 1-Dodecene treatment, using transcriptome and differential transcription analyses, to determine the specific molecular mechanism underlying this olfactory response. Notably, we identified only 53 DEGs that met our screening criteria, and further screening revealed that that the attraction of *C. cunea* adults to 1-Dodecenemay involve transmembrane signal transduction and protein dephosphorylation.

Although no chemosensory DEGs met our initial screening criteria, RT-qPCR identified the up-regulation of CcOBP7, CcOBP18, and CcOR2, which are uniquely or primarily expressed in the male and female antennae^13^, after 1-Dodecene stimulation. Therefore, we speculate that 1-Dodecene may bind to CcOBP7 and CcOBP18, which are transported to the olfactory sensory neurons via the lymph and subsequently bind to CcOR2. This binding activates the downstream signaling pathway and ion channels on the cell membrane, which converts the chemical signals into electrical signals that are transmitted to the advanced nervous system in the brain. Once in the brain, this system integrates all odor signals and triggers specific behavioral responses^38^.

Our study revealed 1.73- and 1.7-fold increases in the expression of CcCSP4 and CcCSP10 after 1-Dodecene treatment relative to the control, respectively, suggesting that these CSPs, together with CcOBP7 and CcOBP18, may regulate the olfactory response to1-Dodecene. Moreover, the expression of the taste receptor CcGR18 also increased after 1-Dodecene treatment, although the participation of this receptor in the olfactory response to1-Dodecene remains to be studied.

In conclusion, the insect olfactory response is rapid and sensitive. Usually, the process by which the olfactory receptor receives and converts chemical signals into electrical signals to trigger specific behavioral responses in insects requires only a few minutes, and this short duration may not enable significant changes in gene expression. However, key proteins in the related signal transduction pathway may undergo modification, especially phosphorylation, during this process. The 53 DEGs identified in a comparison of transcripts obtained from *C. cunea* before and after 1-Dodecene stimulation suggests that these genes may be early responders associated with host localization and subsequent parasitic processes. Further studies of post-translation modifications are needed to elucidate more thoroughly the molecular mechanism underlying this biological process.

## Materials and Methods

### Ethics statement

*C. cunea* is a common insect species and is not included on the “List of Endangered and Protected Animals in China.” All experiments were performed according to ethical guidelines with the intent to minimize pain and discomfort to the insects.

### Insect rearing and RNA preparation

Parasitoid wasps (*C. cunea*) were obtained in 2017 from the Natural Enemy Breeding Center of Luohe Central South Forestry (Henan, China), and they had been cultured in our laboratory at Tianjin Normal University (Tianjin, China) for 7 months. The insects were reared at 25 °C with 70% relative humidity and a 14-hour/10-hourlight/dark cycle. The detailed rearing methods were published previously by Zhu *et al*.^12^. For PacBio sequencing, a total of 100 *C. cunea* adults were immersed in RNA Later (Ambion, AM7020) and collected in RNase-free Tubes (1.5 mL). For Illumina sequencing, one-day-old female *C. cunea* adults were exposed to 1-Dodecene or not for 1 hour, and then immersed in RNA Later (Ambion, AM7020) and collected in RNase-free Tubes. Each tube contained 100 individuals (control or 1-Dodecene treatment) which constituted a unit sample. A total of six unit samples were divided into two groups (control and 1-Dodecene treatment) with each group containing three duplicates. All tubes were stored at −20 °C until processing. Subsequently, total RNA was extracted using Trizol reagent according to the manufacturer’s instructions. The concentration and quality of RNA were determined using a Nanodrop spectrophotometer. The integrity of the RNA was detected accurately using an Agilent 2100 device (Tables S3 and S4).

### Library construction and PacBio sequencing

First-strand cDNA was synthesized using a SMARTer™ PCR cDNA Synthesis Kit (Clontech, Palo Alto, CA, USA) according to the manufacturer’s instructions. After subsequent PCR amplification, quality control, and purification, the cDNA products and the Pacific Biosciences SMRTbell Template Prep Kit were used to construct a library. Qubit2.0 was then used to quantify the library accurately, and Agilent 2100 was used to ensure that the library size was consistent with expectations. Finally, the library was subjected to full-length transcriptome sequencing on the PacBio RS II platform by Beijing Biomarker Technologies Co., Ltd (Beijing, China).

### Transcriptome assembly

The PacBio SMRT sequencing platform (PacBio RS II platform) was used in this study. Full-length transcriptomes were acquired through a 3-step process^39^. In the first step, raw reads was processed into error-corrected reads of insert (ROIs) using the Iso-seq pipeline with a minFullPass of 0 and minPredicted Accuracy of 0.80, and full-length, non-chimeric (FLNC) transcripts were identified by searching ROIs for poly-A tail signals and 5′ and 3′ cDNA primers. In the second step, ROI sequences from the same transcript were clustered using an iterative isoform-clustering (ICE) algorithm. Similar ROI sequences were clustered together to yield consistent isoforms, and full-length (FL) consensus sequences from ICE were polished using Quiver. High-quality FL transcripts were defined as those with a post-correction accuracy >99%. In the third step, non-FL sequences were used to polish the newly obtained consistent sequences and yield high-quality sequences for subsequent analyses. In this step, the accuracies of low-quality FL transcripts were improved by proovread software using the corresponding Illumina RNA seq data and proof read software. CD-HIT^16^ software was also used to merge highly similar sequences and remove redundant sequences from high-quality transcripts to yield a final set of non-redundant transcript sequences. Clean data in PacBio sequencing procedure was kept if the sequences with polymerase read fragment lengths >50 bp and a predicted consensus accuracy >0.80. Next, the rest of the sequence was broken from the adaptor and then the subreads could be obtained with the adaptor sequence filtered out. Only the obtained subreads with the fragment length over 50 bp could be finally considered as clean data.

### Illumina sequencing

The NEBNext UltraTM RNA Library Prep Kit (E7530L) for Illumina (NEB, USA) was used to construct the Illumina library. Briefly, polyadenylated RNA (mRNA) was isolated using Oligo (dT) beads and fragmented into approximately 200-bp fragments using fragmentation buffer. First-strand cDNA was synthesized using random hexamer primers. After second-strand synthesis, the resulting cDNA was purified using AMPure XP beads and subjected to terminal repair, A-tail addition, and sequencing adapter linkage. After size-selecting the purified and repaired double-stranded cDNA fragments, PCR enrichment was performed to obtain cDNA libraries. Qubit 2.0 was used for the preliminary quantification, and Agilent 2100 was used to detect the library insert sizes. Further experiments were performed when the insert sizes met expectations. Each library was checked, and effective library concentrations (>2 nM) were quantified accurately using q-PCR. Different libraries were pooled according to the target volume for offline data and sequenced on the Illumina HiSeq platform. Clean data in Illumina sequencing was obtained by removing reads containing adapter, reads containing ploy-N and low quality reads from raw data.

### Alternative splicing and and SSR detection

The non-redundant transcript sequences were directly used to run all-vs-all BLAST with high identity settings. BLAST alignments that met all criteria were considered products of candidate AS events: (1) both sequence lengths exceeded 1,000 bp and the alignment contained 2 high-scoring segment pairs (HSPs); (2) the alternative splicing gap exceeded 100 bp and was located ≥100 bp from the 3′/5′ end; and (3) a 5-bp overlap was allowed for all alternative transcripts. Transcripts longer than 500 bp were screened and subjected to a SSR analysis using MISA software.

### Detection of CDS and long non-coding (lnc) RNAs

TransDecoder (https://github.com/TransDecoder/TransDecoder/releases) was used to identify candidate coding regions within transcript sequences. A combination of 4 computational approaches with the power to distinguish protein-coding genes from non-coding genes, namely the coding potential calculator (CPC)^26^, coding-non-coding index (CNCI), coding potential assessment tool (CPAT)^27^, and Pfam, was applied to the transcripts to sort the candidate non-protein-coding and putative protein-coding RNAs. The latter were then filtered using minimum length and exon number thresholds. Putative protein-coding RNAs were filtered out using a minimum length and exon number threshold. Transcripts with lengths more than 200 nt and have more than two exons were selected as lncRNA candidates and further screened using CPC/CNCI/CPAT/Pfam that has the power to distinguish the protein-coding genes from the non-coding genes.

### Annotation

BLAST^25^ software (version 2.2.26) was used to compare the newly obtained non-redundant transcripts with those in the NR^40^, Swissprot^18^, GO^19^, COG^20^, KOG^21^, Pfam^22^, and KEGG^23^ databases and thus obtain annotation information.

### Differentially expressed gene analysis

The next-generation transcriptome sequencing was performed with 6 samples, being separated into the control group and 1-Dodecene treatment group with 3 replicates per group. Subsequently, RSEM^41^ software and Mapped Reads location information about the non-redundant transcripts were used to quantify transcript expression. Fragments Per Kilobase of transcript per Million fragments mapped (FPKM) was then used as an index to measure the transcript or gene expression level, and DESeq.^42^ was used to analyze differential expression levels among samples, with a fold change ≥2 and false discovery rate (FDR) <0.01 as screening thresholds. To reduce the prevalence of false positives, the Benjamini–Hochberg correction method was used to correct the p-values obtained using the original test for significance. Finally, the FDR was used as a key index for screening differentially expressed transcripts.

### Real-time quantitative polymerase chain reaction (RT-qPCR) analysis

One-day-old *C. cunea* adults were exposed to1-Dodecene for 1 hour. Total RNA was then extracted from the insects using Trizol and used as a template for the synthesis of cDNA together with TransScript First-Strand cDNA synthesis SuperMix (TransGen, Beijing, China). There were 22 genes subjected to RT-qPCR, including 9 CcOBPs (OBP6, OBP7, OBP14, OBP15, OBP16, OBP18, OBP19, OBP26 and OBP27), 3 CcCSPs(CSP1, CSP4 and CSP10), 7 CcORs(OR2, OR19, OR22, OR45, OR47, OR54 and OR76), 2 CcGRs (GR2 and GR18), and 1 CcSNMP (SNMP1).The specific primer pairs used for RT-qPCR were designed with Primer 5, and the primers used for this study were shown in Table S8. GADPH was used as the internal controls. RT-qPCRs were run using a Roche LightCycler 480 (Stratagene, La Jolla, CA, USA) with the following the cycling parameters: 94 °C for 30 sec, followed by 40 cycles of 94 °C for 5 sec, 55 °C for 10 sec, and 72 °C for 10 sec. Subsequently, the PCR products were heated to 95 °C for 5 sec, cooled to 60 °C for 1 min, heated to 95 °C for 30 sec, and cooled to 50 °C for 30 sec to measure the dissociation curves. The Ct value of each reaction was calculated using Roche qPCR software, and the relative expression level was determined using the 2^−ΔΔCt^ method^43^. All data were normalized to endogenous *GAPDH* levels in the same individual samples. Each sample was analyzed in triplicate, and correlation analyzes were calculated using the Pearson test and SPSS software.

## Supplementary information

Dataset 1

Dataset 3

Dataset 4

Dataset 5

Dataset 6

Dataset 7

Dataset 8

Dataset 9
