# Supplementary material for: Full-Length Transcriptome Survey and Expression Analysis of Parasitoid Wasp Chouioia cunea upon Exposure to 1-Dodecene
Source: Sci Rep. 2019 Dec 3;9:18167. doi: 10.1038/s41598-019-54710-0 (PMC6890788; doi:10.1038/s41598-019-54710-0)
Supplement: Supplementary file 2 — Dataset 3 [file 41598_2019_54710_MOESM2_ESM.pdf]

Full-Length Transcriptome Survey and Expression Analysis of Parasitoid Wasp *Chouioia cunea* upon Exposure to 1-Dodecene

Lina Pan[1], MeiqiGuo[1], Xin Jin[1], Zeyang Sun[1], Hao Jiang[2], Jiayi Han[1], Yonghui Wang[1], Chuncai Yan[1], Min Li[1]*

[1]Tianjin Key Laboratory of Animal and Plant Resistance, Tianjin Normal University, Tianjin 300387, China.

[2]South China University of Technology, 381 Tianhe Road, Guangzhou 510641, China.

***Corresponding author:** Min Li, Tianjin Key Laboratory of Animal and Plant Resistance, Tianjin Normal University, Tianjin 300387, China. E-mail: skylimin@ tjnu.edu.cn. Tel: +86 022 23766673

Table. S2 The nucleotide sequences of 9 OBPs, 4 CSPs, 7 ORs, 1 IRs, 1 SNMPs and 15 GRs of *C. cunea* identified in present study.

>CcOBP26 [ORF 363 bp] Accession Numbers: MN616829

ATGAAACTTTTACTTAGTCTTTGCGTAGTAGTATGCGCCGCTATTGTTAATGCAACAGCA
CCAGAGGATGTAGCGCAGTGTGCTCGCGATACTAATTCGAATTTAGAACAATTGAATGC
TGTCAAAATAGACGACAATTTTAGCCCATCTGAAACGATGAAAAATTTTGCTCTGTGTG
TTTACAAGAAAGCAGGAATCATGAACGAAGATGGGACGCTAAGTAAGGAATTCGCTGA
AGGGGAGCTAGCCAGCATCTGTAAAATTGTGGCCGCCGGAAAAGGTGAAACCGAACT
TGTTGAAAAGCTGATCAAATGTTTTGCGCAACATAACGACCTGGCTGACGGTCCCGCA
AAGTTAGAATAA

>CcOBP27 [ORF 387 bp] Accession Numbers: MN616830

ATGAAACTTTTTCTTGCTCTTTGTGTGCTCATGGGCATCGTCAATGTTTATTGCGAGGAA
ACTTTGAAACTGGCGAAAAATATAAAAGCACACAATAATGATGTCGCGGACTGCGCTA
AGAGAAATAAAGTCGATATAAAAATATTAGACGAGATAAGTAAAACCAAAACTGTTCC
GAAAACCCCTGAGATACAATGTTTTATGGCTTGTATGATGAAGAAAAATAATTTGATGA
ATGAAGATGGAACGTTATCTAAGCAATTTACTAATACGAAAGAACTTGAAATTTGCAAA
GATTTGAGTGGGAAGGACGAATGTGAAATCGCGTTTAAAGCGGTAGAATGTTTCTTGG
AGAACCACCTCATTGAATTAAATAAATCAGATTGA

>CcOBP28 [ORF 402 bp] Accession Numbers: MN616831

ATGAACATTCGCCCGTGTATAGCCGGTATTTTGTCCGTTCTAGTATTAGTGCAGTGCGAA
ACTTCGACGAACGACGATCGACAGTTAGAAGAGTGTCTAAAAGAAATTGGAGCGACC
AAAGACTCGTTTACAGGCCCACCGAATTATGACGACCCCAAGGTGAAATGTCTACAAG
CTTGCTACTTGAAAAAAAAATGGAATGTTAATCGATGGCAAGGCAGTGGCTGACAAACT
TATAGACACGATGTCAAAGGACAGACCTAAAGACGTTATCGATCTATTAAAGGCATATA
TTCCCGCGTGCGTCGATAAAGCTAACGAAGAAAAAGACGAGTGCGAACTGGGTGAAA
TTCTTCGCAAGTGTATCCTGCGGAAAATCCCTCACCTCGCTTCGCAACATTGA

>CcOBP29 [ORF 393 bp] Accession Numbers: MN616832

ATGCGTGTTTTATTTGCTGTTTGTACAACAATCTGCGTCGTTGCAGTTTATTCCAGTAAC
GATGCTGCATCTAAAATTGCTAAAGGAAAGGAAAAACCCAAACAACTAAACGAGGAA
TGTGCTCAAGAAAATGGATTCAACTTGAAAGCAATGTACCATGAGATAGCAACGCATA
AACATTTTGTTGCAACTCAACCAATACGCTGTTTTGTTTTTTGTATGTACAAGAAAATTG
GACTTATGAACGAAGACACGTCGTTGACCCAAAAATTTTACGATACGGAGGGTGCACA
TAAATGTACGCATATAAGTGGGAACGATGAATGCGATACCGCGTACAAAATAATGGAAT
GCGTAACCAAACATGAAATATGGTTTAAAGTGGACTATTAA

>CcOBP30 [ORF 393bp] Accession Numbers: MN616833

ATGAAAATCTACGTGTGCGTTGCAGGCGTTTTGTCCGCTCTTGTACTCGTTCAATGCGA
AATTGCATCCGAATTGAAGAGTCAACTCGACGAATGTCTCAAAGAATTTGAATTATCCG

AAGAGTCGTTAAAGTCGCCCAATTTCGAAGATCCGAAAGTTCAATGCTTTGCTGCGTG
CGTGATGAAGAAAGGCGATCGTTTAGTCGAGGGCAAAGTATCGATTGACAAGGAAGT
GGAACTTGTTCCAATGTACACGCCGAAAAAGATCGACGATGCTTTAAAAGAACAATTC
ACCGAATGTGTAAACAAAGCTAACGAAGAAAAAGACGAATGCGCGGTGGCGGGCTCT
CTGTACAAATGCCTGTTTGAAAAATATGGAGCTTTGGCCTCGTAA

>CcOBP31 [ORF 390 bp] Accession Numbers: MN616834

ATGAAATTATTTATTGTTTGTACAATAGTTGGTATCGTTGGTGTTTCCTCCCACAGCATAC
AAGATAAATCTAAAGTCGACGGTGAGATAAAAAAATTGCCAACAGAAGTGGAACAAT
GTGCTATTGAACAAGGACTTGCAAATATAAACGATGTTGAGGAAACTATAAAAAATAA
AGATTATAACAACTTGACTCGAGAAATGCGTTGCTTTACGGCATGTGTCTTCAAAAAGC
AAGGACTGATAGGCCCCAATGGAACAGTACATAAGAATTTTCAACGACCGGAAATAGC
TAAACACTGTAAAGACTTGAGTGGAAAAGATGAATGTGAAATTGCTTTTAAAATTGTA
GTGTGCATGAACCGACCAGAGTTTTTTACAGATGATTGA

> CcOBP32 [ORF 435 bp] Accession Numbers: MN616835

ATGAAATCGCTTGTTCAAATAGCGCCATTTGTTCTAATTTTGCTAAGTTTTGAGACTGTC
AATGCCAAAATGACAATGGATCAAATTAAGAATACTTTGAAGCCGTTTAAAACGTCGTG
TCTTAAAAAAACTGGAGTGGATATTGACTTAGTAGACGGAACTAAATCCGGTCATTTCC
CGGAAGAAAGATCTTTAATGTGTTTTACAAAATGCGTTATGCAAATGATGAAAGTGGCA
AAAAACGGTGAAATTTTGATAAATGCAATGATGCAACAAGTAGACTTAATGATGCCCGA
TGAGTATGTCGATGAAATGAAATCGATCATTACCAACTGTGGACCGGAAGCCAATACAA
AGGACGATGGTTGTGAATCAGCCTTTACTTTTGCCAAGTGTTTCTATCAATCAAATAGT
GACATCTACTTTTTCCCGTAA

>CcOBP33 [partial 480 bp] Accession Numbers: MN616836

GGCTATTTTAGAACTCAGTTTCGAATAGTACAGTCGCCAAGTTTGCTCACTATACGTCA
AAAGTCTTTACGCATACTCAACATGAAAATATTACTTGTCATTCTCACGTTTTCCATCGT
TGTTGTTGTTTATTGTCACGATGGTCATAATCATTCAAAGATAGTTGAGATACGAGCGTC
GAATCTAGATCTGGAAGACTGTGCTCAAAAACATAAAATTGATAAAATGACACTCAAC
GAACTGAAGAAGAATAAGATTTTCACTGCAACTATAGATACGCAATGTTTTGCCTCTTG
CATATTGAGAAAAACTGGAATCATGTTAGCCGATGGAACGATAAACAAAGATTTCGTTG
ATTCGGATATCGCGTCCGAATGTAAAAACAAGGTTATTGCATTTGGAAAAGACGAATGT
GAGATTGCTGCTAGAATTTTGGGATGTCTAACACAAAAAAATATTATTGTCTTAGCAAA
AGATTAA

>CcOBP34 [partial 426 bp] Accession Numbers: MN616837

AAGCCTCGCGATGAATACCTTGACAGGTTTCTGCTGCTATGCGCAGTAGTTGCATTCAA
AGTGCAAGCTAATATTCCAAAATCCAAATTTAATCCTGAACTTAAAAATGTGTGGGACA
AATGCACGAAAGAAGCTAAACTCACCGAGGCAGAGTTGGATATTGTATTGGAAAATTA
CACCAAGATTACAGATCAAAGAGTAAAGTGTCTGAAAGGATGTCTTTTTAAATCTCTCC
ACGTTATAAACTCTGATAATAAAATTGACGAAACTGCAGCCCAAAAGTACTACGAAGG
TGTAGCCACTAAAAAAAGATACCATCAACGAGGCAATACAAAAGTGCATTCCGAGAGCG

ATTGGAAACGATCACTGTGACATTGCTCAAAATTTCGAGTCTTGCCTTTGCTCTGAATAT
ATTAAGTGCAGCTAA

>CcOR22 [ORF 270 bp] Accession Numbers: MN616858

ATGGTGTACGTCGTGCAACTGTATCTCTGCCAATGGACCCCGGATCATCTGTTCGACGA
GAGCCTGGCCGTGTCGACAGCTGCTTACTTTGCCTCGGTATACTGGATGCCGCGACGAA
TGGGAAAACATCTGCTAACGATTATAGCAAGAGCTCAGCATCCCGTGCAAATCACCGC
AGGTGGATTCGTCAATCTCTCGCTTCAAAGCTTTGGTTCCATGCTCACCAGTGCCTTCT
CCTTTTTCACTTTACTCCGTAAGTTGAATTTGTGA

>CcOR49 [ORF 762 bp] Accession Numbers: MN616884

ATGGACCGACTGATGCCCCTGCAAAACGAAACGCGCCAACGCATCTACCCCTATCATC
CGGAATATTTCTTCGTGGACAGCGAGCGCTACTTCTTCCAACTGTTCAGCTTCCACGGG
ATCTTCGTCGTGGCGTTTTCCACGCTGGCGGTGGCCGCCATCGACAGCATGATGGTCGC
CAACATCGTGCACGCCTGCGGCCTCTTCGCCATCGTGTGTCACAGGCTAGAGAACATC
GGACGGCGGCCGAGGTCCCGGGAAGACGCTACTGGACTGCGACTGGTCGACGATCGC
GACGTTTACCGGCGCTGCCTCGAGGCCTGCCAATTGCACTTGTCCTCCTTAAATAGCGT
CCGCGGAATACAAGAGTCCTTCAGCACGAGCTTCCTGGTGGTGATGGGCGCGGGCGTG
GTGGCCATTGGGATGCTCATGTTCGACTTTCTGTCCAGCCTCGACGAGCCTCTCCGACT
TGTCATCATAGCGGTGCTGCTCTTTGGCATAAACGTATGTCTATTCTACTTGAACTGGGT
TGTTCAGCAAGTGATCGACAGCAGCGAAGAAGTGTTCCAGTACGCCTATTACAGCCAG
TGGTACAACCTGTCGATCAATTCGCGCAAGATGATCATGATCATACTGCAGAACAGCAT
GAAGCCCGTCGCTCTGTCCGCAGCCTCGATATGCGACTTGAATCTGGAGATGTTTGCTT
CCGTTGTCAAAACGTCGATGTCTTACGCTACGGTCATGCTCTCGATGCAACAGCAATAA

>CcOR53 [ORF 1182 bp] Accession Numbers: MN616888

ATGGATTTTCAAAAAAGCGTTGAACATCAATTGTTCGCTATTCCGTTTACCGTGCTCAC
GTTGTGCGGAGCATGGTGTCCAGAAAATTGGTCGCACGAGAACAAACGTATTTACAGT
GTTTACACGTGCTTCATCATGATCTTGGGTATAATTTTTTTCACCGAAATGATGATCAATA
TCGTCTTAACGTTTAACTCGGATAATTTCAACATGGAAAACATATTTACCGCAATTGTCG
TTGGAGTTGGTATTTACAAGAAAATTAACGTATTACTTTATCGCGCAAACATTATGAATT
TTATCAACGAGTACGCGACTAATCAATGGTACAAGCCAAGAAATATCGAAGAAACTGC
CATCTACTCGGCAAACTTGTCCGAACGAAGGCGAGTGACCATCGTTTACGCCGGTCTG
ATCTTGGCGGCTGTTCTACTAAGATCTATATTACCAATACTAGAAGCTGGTACGTTTTTG
ATACTGCCATTGGAAGCTTGGTACCCTTACAACGTGGACAATGTCGTTTCATTTCTCTTA
ACGTACCTTCATCAAATGATCAGCGGTGTGACGCTTTCGTGTATGCATCTCAGCACGGA
TACACTGTTCGTCAGTCTGTTGATGCAAATGTGCTGTCAAATCAATATATTGAAACATCG
ATTGCCAAAAGTTGACGAATCGGGTACTGATAACGACCGTATGAACGAACAAATGTCA
AACGCACGATTTTTGATCGAACAACGCGTGCGCGAACACGAAAGTATTTACAGATTTG
GATGCGATTTGCAAGATACTTTCAGGCCACTTCTAGCGGGTCAAATGGTTATATTCGTAC
CGAATCTCATTATCAATATATATTTTCTTTCCACGAATAGCAACAGGCAGAATTTGAAAT
ACTTTACAACCTTATTTTATGCTTCAGTGTCGTTAATGCAAATTTACATGTTCTGTTGGTA
CGGCAACGAAGTCACTTTGGGTAGTATCGACCTCGGCAATGCCCTGTACGAAAGCAAC

TGGATCAATCTTAATCACCGAACGAAAAAACTACTCTTGACGATAATCACTCGAACATC
CAAGTCTATATTGATATCTGCCGCTGTTATTGTTCCGCTCAACATAGATTCGTTCATCAAA
ATTATGAAAACCTCCTACTCTGCGTTCAATTTTCTCCAACACGTTTCGACTTAG

>CcOR68 [ORF 384 bp] Accession Numbers: MN616903

ATGCGCGTTGTACTGCCGCTTATGTTCTTGAGTTTGATCACGTTCTGTACGAGTGTTTAC
AATGTATCGCAGTCAAAGAATAGTGGCGCCGAGTGGTTTTCGTTCTTTATATATCTCGTG
TGTCTACTTTGTCAACTTTGCTGCTACTGTTGGTTCGGAAACGAGCTCAAACTAAAGA
GTACCGCTGTACGCGAATCTATATACAACAGCGACTGGACTACGTTGAAACCCGTGGA
CAGGAAAAGCTTGTACTTGATTATGCTATCGTGTCAAAGAGAAATGAGCATTTCGTACC
ATGGATTCTGTACGCTTACAATAGACATCTTTGTTTGGGTATTGAAAACCTCGTACGGA
GCTTACAATTTATTGAAAACAGTCGAATAG

>CcOR73 [ORF 528 bp] Accession Numbers: MN616907

ATGAAGGACGTGTTGAATCGGCACAAATCTGCCGAGACAATGGAAGGATGTTCAAGTA
CTTTCTATCTGGAAATTTCTAAAGTTATCAGGTTGCACAAACATGCTTTACACTTTGTAG
ATCTTGTCGAATCGACTTATGCCTCGATGCAGATATTCATCACTGGTCTCACATTGGCGA
CAATTACATTATCGGAATTCGAGGCAGCGGTAAATAAGACACATCAGGATATAAGATTT
CGTTTTATCATCTACGGCGCTGGTGAATTAATTCATATACTATTCCACAATTATCCTGGTC
AACGAGTCCAAGATCACAGTCTCATGATTTATCAATCGTGTTACGACTCTGAATGGTAT
AGAAAAGATGTACCTAATGATTGTAAAAAGCTTATTAATCTGATGATGATAAGAAGTCA
GAAACCATGTTATCTGACAGGTGGAGGATTATTCGTTCTTGGTTTAGAAAATTATGCTAA
TATATTAAAAGCATCTCTATCGTATTTCACTTTCTTGTCTTCAGTACAGTAA

>CcOR80 [ORF 1116 bp] Accession Numbers: MN616912

ATGTTGATTCAGCGGAAGGACTTGTTTGGTTGGAGTGCATTTTTCCATCGTGCGATTGG
ATTATGGCCCGAGGACGATCAATTTCTTGGTGTATATTGGTGGATCAAAAGTTACTTTGT
GATATTTGTTACCGTTGGAGGATTAATTTTTCACTTGTCATCTGTTGCCAATCGAAAGGA
CAACCTCAACTTAACGGAATTTGTTCTTGAGGCTTTGGTAGCAGCTCTTGCGATTGCCA
AAAGTGTGCCAATTGTCGTTAACAGGACAAACGTTTTGAAAATTCTGTCGATGACGCT
AGAATTTAATCTAACGAGTAAAGAAACTTTAAACGAGAAACATATATCAGATGGTTGGT
TCAAGTTGCAAAATAGATTAATAAAATTTTTCATCAGATCCTACACTCTTGTCACCGTCC
TGTATTTTTTGATACCTTGGCTGACGAATCAAACCAATGTATTCCCATTAAAAGGATATA
TGCCCGATTACTTGTACACGACTCCAGGTTTTCAGATTGTCTACTTTATCGAAGTGATCG
TCTATATTATTCGTTTTATTGGCGCGGTGTCCTGCGATCTATATTGCACCACTTTCATCTG
TCAACTATTTAGCGAACTTCAGATTGTTCAATACAAATTGACAACGCTGAAGAAGTGTA
GACAAAGCTTGATTCGTATAATTCAACAACACGCCAAAGTACTCGATCACGGCTATATG
ATCTCGAAAAACTTTTCGATCGTCTTTTTATTGCAGCATGTTTTACTATCGATGATCGTAT
GCTTTTCTGGATTAAATGCACTTTACACGGACAGTTTGGCGGTGATCGTGAAAATGTTG
TCCTTCCTCTCGTTGGCCCTGGTGGAATGCTTTTTCATCTGTTTGATCGGTGAACAAATT
AAAGAGGAGAGCTTAAAAGTTGCCAACAATCTAGAAGATATTTACGACGAATGTTTAG
ACGATCTCGTGATAAGAAAGTTGATCAATTTTGTACACCTTCGCGCCCAAAAACCTCTC
GAGTTGAAATTTGGAAAATATACCGTTATCAATTTACAATTTTACTGTGAAACTCTAAAA
ACGATATTTTCGCTTTCCACGGTATTCAAAACTATGATCAAATAA

>CcOR81 [ORF 1107bp] Accession Numbers: MN616913

ATGTTGAGCGTGCAAAAAAGAGTCAACTTGATAATAATTTCATTGAAAGTACTCGGTTA
CGATCCATCTTCGGTTAACCGAAAGTGGCGCGTAAGTACATTTTTGTGGTGGTTTTACT
TGTTAAACAACGTGATATTATTATTGTTAACAATCTTCACTATGTTTTCGGCAATAAGTGA
TGCCGATAAAGATATCATGACGATGAGTTTGACCACGATTGAATTGAGAACGTTTACCG
AGCTTGTTACTATTTTGTTGAACTACAAATATAAGATCAATCAATTTAGAATCCTAATAC
AGCTGATGAAAAATGACGTAAACGACAAAACTTTTACAGTCGGTTCAAAACATGTAAT
TAAGTACGTAAAAATATCTTTTTTGATATTGGCTCTCTATTTGATCGTAATTTACGTATACA
TAAACAACTTTGAGTGGCAAAATGATAAAAAATCCCTAAGTACGGCAATCTATCCTTTC
AAATTAGATTCGACGTTGAAAAAATTATTAATTTTGACGCATCAACTAGTGATACTCGTG
CACTCGAGCTCAACGTTCATCTTCGACGGAATAATAGCTCTTTTGATATATACGTGTACA
GTGAGACTAATAATATTGAAAGACAACTTGAAAACGAGCAAAGATTCCAAATTTAAGC
AGCTAATTCACGAGCATCAAGAAATTCTTAAATTAATCGACAGTGTTAACGATTTTATTA
GCTACCTCGTTGCTAAAACTGCGTTCAGTTTTGTAAGCAGTGCTATTTCAGCGTTTGTG
CAATTGATCCAACAAAAATCACTGAAAGGCATGGCACTGGAATACTTGATTGTTGTAGC
GTTTGGTTTAAGAATATTGGTTTGCGCGGAGAGTGCTGAAGATTTAGCTGTATGTAACG
AGAATATTCAAAATGAAATTTACTCGTCTCTGTGGTCAACGAAAAACAGAGGGATCGT
CGTTTCAAAACTGATAATGATGCGTAGATGTCAAGGAATACCAAAGATATGTATAAAAG
GTACATTTATATCTACGCTTAACAGAGAGTATATCAAACACATACTCAAAACGATATTTTT
ATACTTCGTAGCTATGAGAGCAATAATGAGCAGGTGA

>CcOR82 [ORF 1188 bp] Accession Numbers: MN616913

ATGGAAAACAGCAAAGCAGTGGAGCATTTAAAAACTCTCGAGACTTTGATGATAAAAG
CTATGGAAGGAAATCGAAGCAATTACAACAAGGACGATTTGGAAAAGTTGTTGAGAA
GTAGCGATCCAGATGACGAGCAAAGACTCAAAACTACAATAATCGACATCTTGGAATG
CGACGACATCGTCGCTATCAAAATATTAATAGCAAATCAAGCGATCGATTTATTCTCAAG
AGATTTCGAGGAAAAAACTGCGTTATCCGTGGCTGTATCCAGTGGTAGTATGGATATCG
TCAAATTATTTTTAAATCAAATGAACGAATATGACGTAATTCGATATAACAATGAACAAC
TTCTTTGCTTTGATTTTCCAAAAATTTTACCAGAATTATATTTAGTTGAAGATATCATATG
GATCGTTCGTATATTGATGTCGAAGAAATTTCTGTTGAGTTTGAAGCACAAATTGGCGG
TTTTGGAATTTCTCGTGCACGATAGAAACGATTGCGATTGTGCTACTGGAGACATAAAG
CAACGTTTAGCTGGATTGATCATGTATGGTTCTGCGACCCACGTCAAAGAGATTATCAA
TAAAATTATGGGTCAAATTGACACGGGAGAAATTGAACTCGACAAGGAAGCCATGATT
ACATGTATTAGAAATGCACTCAAGCCTGGAGTAGTGTTCGATTTGTACATCGACAAGGA
TATTGAAGATTACATAACGAGCATTTTTCCTACTTGGAAGAGCACGTTTTCAGACGAAC
CTGATGATACTACTGAATACAACTGGTTAAAAGAGTACGACATTGAAGATGAGAATACG
AAACTCAGTCTTTTCGACATACTCTGCATGAATCCTCGCGATACTTATTCTTACATATCG
CATCTTAATTTTCATCAAATAACCACGCAACACGCGTCGGCTGGTGAATATATAAAAAG
TCTCAAAGCAAAATCGTTGATTAGACGTCAAATTGAAGAGTTTGTGCCCGAATTTATGA
TAGATTTTTTTCCTCAGTTACCTGAAAATTGTGCCAATCTTGTAATACACTACATACAAT
GTGATTTAAATAATGAAAATATCTTGAACATGTGTCGGATCGCCTTTGCCGAGCTGCGT
AAAAATTGCGATATGGTTGAATCGTATCAAGAATACGTTATCGATATGGAAATTGATAGA
TATTAG

>CcGR6 [ORF 1494 bp] Accession Numbers: MN616771

ATGTACGAAGCAAGAAAAAAGGTTTTTTGGTTTGCTATAGTCTCTCGATTATCAACTTT
GATACTGCAAGTGATTTTCAATGTACTATGTCCAGATCACGATGCAGACGCCTTTCGTA
CACCTGCAGATCCAACTGAAAAACATTCACGTTTAGACAATATCGTAACATTTTTATTAG
AAGGACTGACGAGATGGGATGCACAATATTTTATTCATATAGCGAAATATGGTTACACAT
ATGAAAATACTCTTGCCTTTTTTCCACTTTTTCCATTATCGATGAAATATATGGCCAGAGT
GTTTAGAATACAACCACCAATACTTAATTATAGTAACGTCATTGTGATATGCGGCGTTGT
AATTAATTTTGTATGTTTCGTCAAAGCAGTCTTAGTTTTTTACGATCTCAGCCTAGTAGTA
TTTAAAAACATTAAAGTAGCTTATCGAGCTGCCATATTTTTTTGTGTAAATCCAGCAAGT
ATATTCTTCACTGCTCTTTACACGGAATCGTTGTTTGCTTACCTGTCGTTTTATAGTATGC
TAGAAAGCATCAGCAATAATCCTTGCGTATTTTTGCCATTGAGTCTTTCTAGTTTAGTAA
GATCGAATGGACTAGTCAATCTTGGTTTTCCTATTTATTTTTGGTTGAGAAATTTATTGAT
AACAGTTTTGCCAAACTATGTGTTGGAAAATAGGCATTTTCACGGCAATTCAAAATCAT
TGCTCTTCAACTTTCGACACGTTTTCATAAGTTTATCCCAAATTATTTTTGTAATAGTTTT
GTCCCTGCTACCATTTGGTTATTCACAAGCTTACAATTATACGAAATTTTGTAAACCGGA
ATTAAACGATTCGTTGCTTCCGTATCACGTACAAGAATATGCAATAGGCAATAATATGTC
GTTGCCAGGAGAACATGATTTTTCATGGTGCAATTCCAAATTACCCATTGCATATTCGCA
CATACAACATAAGTATTGGAACGTAGGTTTTCTAAAGTACTACCAATTCAAGCAGATTC
CTAACTTCATTCTTGCTGTACCTGTAATATACTTGATGTTAAAGTGCTGCATCGAATTTTT
TAACGAACACAAATCAAAATTTTTCACTTTGGAATTTTTCACGGGTAAATCCAGAGCGT
CAGACAATATAAAACAATACCCATTAGAAATGTTCGTTTTTGTAGTACACGCTTTATTTT
TGACGATCTTTTGTATTTTCTTTGTACATATTCAAGTGAGTACACGGCTTCTATGCTCAG
CTAGTCCCGTGTTATATTGGTATTGTGCCTTAGCAACTTTGCGAAAAACTAAAACGTCA
AAAAAACTTAAAGAGATTGAATATGAAAGTTCGGAAAATTTATACTCTCGATGGAAAGT
ATTTTTTATTACTCAACAGCACTATCCTTATCAAGAAAAATTAATCCTTGGATATTTTCTA
GGATACTTTGCGATAGGCTGTTTCATGTATGTTAATTTTTTGCCTTGGACTTGA

>CcGR18 [ORF 2559bp] Accession Numbers: MN616781

ATGAAGGGGAAGCAGCCGGGCTTACTTCTCGCCGTAGTGCTGCTGCTGCTACTACTAC
ACCAGCTGACCAGCGGCTCAGTTCACGCTCTACCTCCGCTCATCAAAATTGGTGCGATT
TTCACTCTTGACCAAGCAAACAGCAGCACCGAGCTGGCGTTCAAATACGCCGTGCACA
AGATCAACAAGGACAGGCTGATACTACCGGATACAACGCTTGTCTACGACATTCAATAC
GTGCCTAAGGACGATTCGTTTCACGCATCCAAGAAAGCGTGTCAGCAGGTGAAATTCG
GCGTACAAGCAATTTTCGGTCCATCAGATCCGATCCTCGGTCAGCACATCCACAGCATA
TGCGACGCGTTGGATATACCACACCTGGAGGCCAGGCTGGACCTGGACTCCGAGGCCA
AGGAATTCAGTATAAATCTGCATCCAGCACAAAGTCTCCTCAACAACGCTTATCAAGAC
GTTATGGCCTATTTGAATTGGACACGTGTGGCCATTGTCTACGAAGACGATTACGGTCT
AGTAAAGCTTCGGGAGTTGGTGAGATCACGAAGATCGCAGGACATGGAAATCTATCTG
CGACAAGCAGATCTCGACTCTTATAGGCAGGTCCTGAGCGAGATCAAAGCCAAGGAAA
TACGCAATCTTATCGTCGACACCAAACCCGGAAATATGCATCATTTTTTGCGGATGATAC
TTCAGCTACAGATGAACGACTACAACTATCACTACCTCTTCACTACTTTTGATATCGAAA

CGTTCGATCTCGAGGACTTCAAGTACAACTTCGTCAACATTACTGCCTTTCGACTGGTG
GACGCTGATGATGCGGAAGTACGTGGCGTCCTTAGGGACATGGAGAAGTTTCAGTTTC
AAGGCAACAACTTACTCAACAAATCACGTGTTATACAGGCTGAGGCAGCACTGATGTA
CGATAGCGTGCAGGTGTTCGCGGTAGGACTACGTACCTTGGAACAGTCGCATGCACTA
AGGCCAATGAACATCTCTTGCGAATTGGAACATCCATGGGATGGTGGACTTTCTCTTAT
CAACTACATCAATACGGTGGACAGGAAAGGGATTTCCGGGCCCATAGGTTTCAAGGAG
GGTCGCCGGATACAATTCAAGCTTGACTTGCTCAAGCTCAGGCAGCAGTCGTTGGTTA
AAGTGGGTGAATGGAGACCAGGGTCAGGGGTTAACATTACAGACAAGTCGGCATTCTT
CGAAGCCGGAACCACCAATGTCACCCTGGTGGTCATCACCATTCTCGAAACCCCATAC
GTGATGCTGAGAAGCAAAGGCAACTTTAGTGGAAACGATCGCTACGAAGGCTTTTGTA
TCGACCTGCTCAAGGAAATAGCGCACATGGTCGGCTTCGCCTACAGGATCGAGCTTGT
CCCGGATGGCAAGTACGGTGTCTACGACTACGAGACTGGCGAATGGAATGGTATCGTC
AGGCAACTCATGGACAAGAAAGCAGATCTAGCAGTAGGCTCAATGACGATCAACTACG
CACGTGAGAGCGTCATAGACTTCACCAAACCATTCATGAACCTTGGTATCTCGATTCTC
TTCAAGGTACCAACAAGTCACCCCGCGCGCCTCTTCTCCTTCATGAATCCATTGGCAAT
AGAAATCTGGTTATACGTATTGGCTGCTTACATTCTCGTCTCGGTAACCATGTTCGTAGT
GGCACGTTTTTCACCCTACGAATGGAACAATCCACATCCGTGTCATTCGCAGAATACAG
AGATCGTGGAAAATCAATTTTCGTTGGCCAATAGCTTCTGGTTCACCATTGGTACTCTAA
TGCAGCAGGGTAGCGACCTCAACCCAAAGGCGACAAGCACGAGGATCGTTAGTGGAG
TCTGGTGGTTTTTCACCCTCATCATAATCTCCTCGTACACAGCTAATCTCGCGGCTTTTC
TCACCGTCGAGCGCATGATAACGCCCATTGAGAATGCCGAGGATCTTGCGAGCCAGAC
TGACATCTCTTATGGGACACTCGAGAGCGGGAGTACTATGACTTTTTTTAGGGATTCAA
TGATCGAGACGTACAAGAAGATGTGGCGATTCATGGAAAATAAAAAACCTTCTGTATTC
GTTCCTACTTATGAAGAGGGCATTAAGCGAGTTCTTCAAGGGAATTATGCCTTCCTCAT
GGAATCAACGATGCTCGACTATATCGTTCAGAGAGACTGCAATCTTACACAAATTGGTG
GCCTATTAGATAGTAAAGGTTACGGTATAGCTACGCCCATGGGCTCGCCTTGGAGAGAC
AAAATTTCGCTGGCAATTCTAGAGCTGCAAGAAAAAGGCGAGATTCAAATTTTATACG
ACAAATGGTGGAAAAGTCCAAGTGACACTTGTATGAGAAATGACAAAGACAAAGGAA
GCAAGGCTAATGCTTTGGGTGTCGACAATATAGGCGGGATTTTCGTGGTATTGTTGTGC
GGATTAACATTCGCTGTGCTCATTGCCATTTTCGAATTCTGTTACAATTCCAAAAGAAAT
GTACCTGTTGAGCGCCATGATGATTTCGACGTCGACTAG

>CcGR19 [ORF 1137 bp] Accession Numbers: MN616782

ATGAATCCACCGTACGCGACCAAAGTCAATCATTCGAGAAGCGTGGAGGGCAACGCG
CGTTACGAAGGATTCGTCGTCGACATCGTCAAAGCTCTAGCCAAAGAGATGCACTTCA
ACTACACGTTTTACGTGCAGGAAAATTCGGACAACGGCAACTGCACGAAGGACGAGA
CGACCGAACAATGCAGCTGCACTGGCATGATGGGCAAGATACTCAGACACGAAATGG
ACATGGCGATAACGGATTTGACCATCACGGAAAATCGCGCCAAGTGCATTCAATTCTCA
ACGGCGTTTTGGAATTTGGGCATGAGTATTCTCTACAAAAAGCCACAGAAAGCCGAAG
CAAGATGGTACTCGTTCTTTTTGCCGTTCTCCAATCGCGTTTGGATGTATCTAGGTCTCG
TGTGGATCTTCACGTCGATACTCTTCTTCGTACTTGGCCGCCTCAGTCCGTCCGAGTGG
ACCAATCCTTTTCCTTGCATCGACGAACCCGACGAATTACACAATCAGCTGACGATCGA
CAATGCCTTTTGGTTCACCGCCGGCGCCATTATGCAGCAAGGTTCCGAGATCGCGCCAA
TAGGAATGTCAACGAGGTGTTTAGGAGGATTTTGGGCGTTCTTTTGCTTGATCATGGTT

AATACGTATATCGCCAATTTGGCCGCTTTCCTGACCATCGAAACACCCGTCAAAGTCGT
CAGGGGTATCGACGATCTGTACAATCAGACGACCATTAAATACGGAGCTAAGAAGGGC
GGTTCCACGTTTACGTATTTCAAAAGCTCGAGTGATCCTAAGCACAAGCAGTTGTACAA
TAACATGATAGACCCGGAATGGATGAAAAAATGGATGGTCGATACGAACGAGAAAGGT
ATCGACTTGGCTAAAAGCGATCAAGTCAATTACGCTTTCTTCATGGAGTCGCCGTCCAT
CGAATACGTGCAACACCGAATATGCAATCTCGAACAGGCGGGTGGGTTGATCGACCAA
AAAGCTTACGCGATCGGCTATGCCAAAAATTTCAGATACATCAAGGAAGTCAATCAAAT
GATATCTCAACTCAATGAGAATTCCGTCATCAAAGAGCTGTACAAAAAGTGGTGGACC
GAAAAAGGCGCCGTTTGCGGGGTATAA

>CcGR20 [ORF 3108 bp] Accession Numbers: MN616783

ATGAATCGGTGGTTATCGATACTCGGATGGCTAACCGTAATGAGCCTTGCCGTTGGTAAT
GAATTGGAAGAAGAACATCCATTATCGGACGACGCAGGTGGTTTTGGTAGCTATGGTG
GAGGTGGAGCTGGTGTACAACGAACAAGTAGTACCATTAAAATCGGTAAATCCGATCG
AAGTAGTGGTAGTCTTGGTAGTACTACTGGTCGCGGCAGTTCTATGATTAAAATTGGCG
AAGGTTCGAAACTACTCAATACAAGTCGTGTGCTTAGGCCAGCTCTTACAACCTCGAT
GACCAGTACTAGTATACCTCTGTCTTACGCATCCGTCCAGCCTGAAAACCAAGGCAATG
CCGAGTTTACCAATGGCACTAAGATGCTCAAGGTAGGTTTAGCAGTACCGTACAAGTC
GTTTGGTTACCGTGAATACACAAAAGCGGTAAGTCGCGTGGTGACAGCTTTGCAAAAA
AGTACCAAACGACCAAATCTTGGACTCTTTCAACATTATGACATCTTCGTAAAAGTAGC
AATGCAAGAATTAACACCTAGTCCAATGAACATCTTGAACTCGCTATGTAAGGAGTTTC
TATCGCTCAACGTATCGGCAATCTTATATTTGATGAATTACGAGCAGTATGGACGCAGTA
CGGCTAGTACCCAATACTTTTTACAATTAGCAGGTTACTTGGGTATACCCGTGATCGCTT
GGAACGCTGATAATTCTGGACTTGAACGACGCACATCTCAGAGTAGTTTACATTTACAA
CTGGCACCATCGATCGAACATCAAGCTGCGGCAATGCTAAGTATTCTTGAGCGTTACAA
GTGGCATCAATTTAGCGTGGTCACCTCTCAAATAGCTGGCCACGACGACTTTGTTCAAG
CAGTACGCGAACGTATATCCGACATGCAGGAACGATTCAAGTTTACTCTTCTCAATGCA
ATTACCGTAACTAAATCGCAAGATCTTAAAGATCTTGTTAACGTCGAATCACGAGTCAT
GTTACTCTATTCGTCAAAAGAGGAAGCTAATAATATCTTTAGAGCTGCTGCTGAATTTAA
GATTACTGGTGAAAATTACGTATGGGTGGTTACTCAAAGCGTCATTCAAAATATACAAC
CTGGACATCACTTTCCTGTTGGCATGATCGGTGTACACTTCGATACGAGTAGTACAAGC
ATATTAAACGAGATAGCAACGGCGATCAAGGTATATGCTTATGGAGTTGAAGACTTCGT
TAACGACCCAAGGAACTATGGCTATAGCCTCAACACTCAACTAAGTTGCGAGGATTTA
AGCAGCGAGTCACGATGGAGCACCGGTGAATATTTTTTCAAGTACTTAAAGAACGTGT
CCGTGGAAGCGGAATACGGCAAACCACCCGTAGAGTTCACTCAAGATGGTGTACTAAA
GTCTGCCGAGCTCAAAATCATGAATTTACGACCTGGAGCCAGTATGCAATTAGTCTGGG
AAGAGATTGGTACATGGAAGTCTTGGGAGAAGGATGGTTTGGATATAAAAGACATAGT
ATGGCCGGGTAATACACATACACCACCACCAGGAGTTCCAGAGAAATTTCACGTGAAG
ATCACTTTCCTAGAGGAGCCACCCTACATCAATCTCGCACCGCCAGACCCAGTGACAG
GCAAGTGCCTTGTCGAGCGAGGCGTTCATTGTCGCGTTGCCAAGGATCCCGATGAGCT
GGATATTCAAGCTGGTGCACGTAATGGCACTGCCTTTCAATGTTGCAGTGGTTTCTGCA
TAGATTTGCTACAAAAGTTTTCCGAAGAAATGGGATTTACTTACGAACTGGTTCGCGTT
GAAGATAATAAATGGGGTACACTCGAGAATGGTAAATGGAATGGATTGATGGCAGAGT
TGGTAAACCGTAAGACAGATATGGTGATGACATCGTTGAAGATTAATTCGGAACGGGA

AGCCGTAGTGGATTTCACAGTGCCGTTCATGGAGACAGGTTCGGCTATTGTTGTAGCAA
AGCGTACTGGAATCATTTCACCCACAGCCTTTTTGGAGCCGTTCGACACTGCTTCCTGG
ATGCTCGTTGGCTTTGTGGCAATACACTCGGCAACATTCATGATTTTCCTCTTCGAGTG
GCTCTCGCCATCTAGCTTCGGCATACCCGATTATTCCTCGGCCACGAAATCAAAGCCGC
AACATCATCGACATCGTCATAATCATTATCACCATTATCGTCACGAGCGTGAACGTCGTC
AAGAACATCGTCAAATAGTTAAACCACCAAGACAACATCGATTCTCCTTGTGTCGCGT
CTACTGGTTAGTTTGGGCGGTGCTTTTTCAAGCCGCCGTCCACGTCGACTCACCAAGA
GGATTTACAGCCAGGTTTATGACGAACGTCTGGGCAATGTTCGCCGTGGTATTCCTTGC
CATTTACACAGCCAATCTTGCAGCTTTCATGATAACTCGCGAAGAATTCTTTGACTTTAC
TGGAATTGACGATCATCGTCTTGCAAGACCTATGTCACACAAACCACCTATTAAATTTG
GTACCGTACCATTCACGCATACCGATAGTATACTTGCCAAATACTTCAAGGAGATGTATG
CTTACATGAAGAATCACAATAAGAATAGCGTATCCGAAGGCATCGAAGCTGTTATCAAT
GGTGACTTGGATGCTTTTATCTACGACGGAACTGTAATTGACTATTTAGTCTCACAGGAT
CAAGATTGTCGACTATTAACCGTTGGTTCATGGTACGCGATGACGGGTTACGGATTAGC
TTTTCCACGTAATTCACGTTTTCTCAAAATGTTCAATCAAAAGCTTCTCGAATACAGAG
ACAACGGTGACCTGGAAAGACTACGTCGTTTTTGGATGACAGGTACCTGTAGACCCGA
TAAAGAGGTACAAAAAAGTAGCGATCCTCTTGCTCTTGAGCAATTCTTAAGTGCTTTCT
TAATGTTAATGGTCGGAATTCTCATTGCCGCAATTCTACTACTTCTCGAGCATATCTATTC
CAAATACATTAGACGTCGTCTCGCCAAAGATAGTCGGGCGAGTAAGTGCTGTGCTCTTC
TCAGTGTCCGAGAAATGATGAATCCCGAGATGGAAATCGAAAGTTAA

>CcGR21 [ORF 1239 bp] Accession Numbers: MN616784

ATGGCAGAGTTGGTAAACCGTAAGACAGATATGGTGATGACATCGTTGAAGATTAATTC
GGAACGGGAAGCCGTAGTGGATTTCACAGTGCCGTTCATGGAGACAGGTTCGGCTATT
GTTGTAGCAAAGCGTACTGGAATCATTTCACCCACAGCCTTTTTGGAGCCGTTCGACA
CTGCTTCCTGGATGCTCGTTGGCTTTGTGGCAATACACTCGGCAACATTCATGATTTTCC
TCTTCGAGTGGCTCTCGCCATCTAGCTTCGGCATACCCGATTATTCCTCGGCCACGAAAT
CAAAGCCGCAACATCATCGACATCGTCATAATCATTATCACCATTATCGTCACGAGCGTG
AACGTCGTCAAGAACATCGTCAAATAGTTAAACCACCAAGACAACATCGATTCTCCTT
GTGTCGCGTCTACTGGTTAGTTTGGGCGGTGCTTTTTCAAGCCGCCGTCCACGTCGACT
CACCAAGAGGATTTACAGCCAGGTTTATGACGAACGTCTGGGCAATGTTCGCCGTGGT
ATTCCTTGCCATTTACACAGCCAATCTTGCAGCTTTCATGATAACTCGCGAAGAATTCTT
TGACTTTACTGGAATTGACGATCATCGTCTTGCAAGACCTATGTCACACAAACCACCTA
TTAAATTTGGTACCGTACCATTCACGCATACCGATAGTATACTTGCCAAATACTTCAAGG
AGATGTATGCTTACATGAAGAATCACAATAAGAATAGCGTATCCGAAGGCATCGAAGCT
GTTATCAATGGTGACTTGGATGCTTTTATCTACGACGGAACTGTAATTGACTATTTAGTC
TCACAGGATCAAGATTGTCGACTATTAACCGTTGGTTCATGGTACGCGATGACGGGTTA
CGGATTAGCTTTTCCACGTAATTCACGTTTTCTCAAAATGTTCAATCAAAAGCTTCTCG
AATACAGAGACAACGGTGACCTGGAAAGACTACGTCGTTTTTGGATGACAGGTACCTG
TAGACCCGATAAAGAGGTACAAAAAAGTAGCGATCCTCTTGCTCTTGAGCAATTCTTAA
GTGCTTTCTTAATGTTAATGGTCGGAATTCTCATTGCCGCAATTCTACTACTTCTCGAGC
ATATCTATTCCAAATACATTAGACGTCGTCTCGCCAAAGATAGTCGGGCGAGTAAGTGC
TGTGCTCTTCTCAGTGTCCGAGAAATGATGAATCCCGAGATGGAAATCGAAAGTTAA

>CcGR22 [ORF 2613 bp] Accession Numbers: MN616785

ATGCACCTCATAAGGTTATTAACGTCCATCATTAGTCTACTAGGTATTAAAGCTTTACCA
GAAATAGTCAGAATAGGAGGGTTATTTCACTCATCAGATAGCAAGCAGGAGGTAGCTTT
TAGATATGCAGTTGAAAAAATAAATGGCAATAGAGATATTTTACCGAAATCACGATTAA
GTGCACAGATCGAACAGATAAACCCTCAAGATAGTTTTCATGCTTCAAAAAGAGTATGT
CATTTACTAAAAACGGGAATAGCTGCAATATTTGGACCTCAAAATGCACATACAGCATC
TCATATACAAAGTATCTGCGACACAATGGAAATTCCGCATTTAGAAACAAGGTGGGACT
TCAGATTGAAGAGGGAAGGCTGTCTTGTCAACTTATATCCTCACCCAGCCACATTATCA
AAAGCCTACGTTGATTTGGTGACTGGGTTGGGATGGAAAGGTTTTACTATAATTTATGA
AAATAATGAAGGCTTAGTTCGTTTACAAGAATTACTAAAAGCTCATGGACCAACAGATT
TCCCGATAACTATCAGACAACTCGGAGAGGAATCTAATATTGGTCATGGTTATAGGCCA
CTTCTGAAGCAAATTAAAAATTCTGCTGAATCACACATTATTTTAGATTGTTCAACAGA
CAAAATTTATACAGTATTAAAGCAAGCTCAAGAAATCGGTATGATGACAGACTACCATA
GCTATTTTATAACATCATTGGATCTTCATACCGTTGATCTTAATGAATTCAAACATGGTGG
AACTAACATTACTGCTTTTAGAATTGTGAATCCTGATAAAACGCAAGAGACAGTACAAG
ATTGGATTTTTGGAGAACAACGATATTTTCGAAAACTTGATATTGAACACAATGAGAAA
AATCACACATTCATCAAGACTGAAACTGCATTGATGTACGATGCAGTTTACTTATTTGCG
CGAGCCCTTCACGTTTTAGATGCTTCACAACAAATTGAAATTAGACAACTTTCTTGTGA
TTCATCAGATACTTGGGATCATGGATATTCACTCATAAATTACATGAAAAATGTGGAAAT
GGATGGTTTAACAGGAGCCATCAAATTTGATAATCAAGGATTTCGATCAGATTTCGAAC
TTGATATAATAGAACTTAATACAAAAGATGGCTTAAAAAAAATAGGAAATTGGAACAGT
ACTAAGGGGATTAATTTCACTCGAAGTTATGGAGAAGTCTATACTCAAATAGTGGATAG
TTTGCATAACAAGACATTTATTGTGACTACAATATTGAGTGCTCCATACTGTATGTGGAA
AGAGTCAAGTAAAAAGTTATCTGGTAATGCTCAATTTGAAGGATATAGTGTCGATCTTAT
TCAAGAAATAGCCCGTATTCTAAAATTCAACTACACTATCCGTCTGGTACCCGATGGTC
GTTATGGTTCGTATACTAGAGAATTGAAAGAGTGGGATGGAATGATTAAGGAATTGTTA
GATCAAAAAGCAGATCTAGCTATAGCTGATCTAACAATCACATATGATCGTGAGCAAGC
AGTGGACTTTACAATGCCATTTATGAATTTGGGTATAAGTATACTCTACAGAAAGCCGGT
AAAACAACCTCCAAATTTATTTTCTTTCTTGAGTCCATTAAGCTTAGACGTTTGGATTTA
CATGGCTACTGCATACCTTGGTGTTTCAGTTCTCTTATTTATTTTAGCAAGATTCAGCCCA
TATGAATGGGAAAACTCCAACACGATCAATAATCAAACAACTATGATGGAAAATGAATA
CACACTTTTAAATTCACTATGGTTTACTATAGGATCACTTATGCAACAAGGATCTGATATT
GCCCCAAGAGCGATATCAACACGGATGGTGGCTGGCATGTGGTGGTTTTTCACACTAAT
TATGATATCATCATATACAGCAAATCTTGCAGCTTTTCTTACAGTAGAACGTATGGACTC
TCCAATTGAAAGTGCTGAAGATTTAGCTAAACAAACTAAAATTAAGTACGGCGCATTAA
AAGGAGGAAGCACCGCAGCTTTTTTTCGCGACTCCAACTTTTCGACTTATCAACGAAT
GTGGCATTTCATGGAAACTGCAAAACCTCCCAATGAGGTTTTTACAAAAAGTAATGTT
GAAGGCGTCGAGCGAGTTGTCAAAGGAAAAGGAAGTTATGCATTCCTAATGGAATCTA
CATCTATTGAATATGTCATAGAAAGAAACTGTGAATTAACTCAAATTGGAGGACTTTTA
GATTCAAAAGGGTATGGAATTGCCATGCCTCCAAATTCTCCTTATAGAACAGCTATTAGT
GGAGCTATTTTGAAACTGCAAGAGGAAGGTAAACTCCATGTTCTAAAAACTAAGTGGT
GGAAAGAAAAACATGGAGGAGGTGCATGTCGGGATGACGCATCAAAGAGCACCTCTA
CAGCTAGTGAATTGGGTTTGGCGAATGTAGGTGGAGTATTTGTTGTATTAATGGGCGGA
ATGGGAGTTGCTTGTGTGATAGCAGTATGTGAATTTCTCTGGAAAAGTAGAAAAATTGC

TGTAGAAGAACGGAGACAACGTTCATCTGAAAAGCCAATGTGCATTGGATTTACCGAT
CTACACTGA

>CcGR23 [ORF 2190 bp] Accession Numbers: MN616786

ATGCACCTCATAAGGTTATTAACGTCCATCATTAGTCTACTAGGTATTAAAGCTTTACCA
GAAATAGTCAGAATAGGAGGGTTATTTCACTCATCAGATAGCAAGCAGGAGGTAGCTTT
TAGATATGCAGTTGAAAAAATAAATGGCAATAGAGATATTTTACCGAAATCACGATTAA
GTGCACAGATCGAACAGATAAACCCTCAAGATAGTTTTCATGCTTCAAAAAGAGTATGT
CATTTACTAAAAACGGGAATAGCTGCAATATTTGGACCTCAAAATGCACATACAGCATC
TCATATACAAAGTATCTGCGACACAATGGAAATTCCGCATTTAGAAACAAGGTGGGACT
TCAGATTGAAGAGGGAAGGCTGTCTTGTCAACTTATATCCTCACCCAGCCACATTATCA
AAAGCCTACGTTGATTTGGTGACTGGGTTGGGATGGAAAGGTTTTACTATAATTTATGA
AAATAATGAAGGCTTAGTTCGTTTACAAGAATTACTAAAAGCTCATGGACCAACAGATT
TCCCGATAACTATCAGACAACTCGGAGAGGAATCTAATATTGGTCATGGTTATAGGCCA
CTTCTGAAGCAAATTAAAAATTCTGCTGAATCACACATTATTTTAGATTGTTCAACAGA
CAAAATTTATACAGTATTAAAGCAAGCTCAAGAAATCGGTATGATGACAGACTACCATA
GCTATTTTATAACATCATTGAAAAATCACACATTCATCAAGACTGAAACTGCATTGATGT
ACGATGCAGTTTACTTATTTGCGCGAGCCCTTCACGTTTTAGATGCTTCACAACAAATT
GAAATTAGACAACTTTCTTGTGATTCATCAGATACTTGGGATCATGGATATTCACTCATA
AATTACATGAAAAATGTGGAAATGGATGGTTTAACAGGAGCCATCAAATTTGATAATCA
AGGATTTCGATCAGATTTCGAACTTGATATAATAGAACTTAATACAAAAGATGGCTTAA
AAAAAATAGGAAATTGGAACAGTACTAAGGGGATTAATTTCACTCGAAGTTATGGAGA
AGTCTATACTCAAATAGTGGATAGTTTGCATAACAAGACATTTATTGTGACTACAATATT
GAGTGCTCCATACTGTATGTGGAAAGAGTCAAGTAAAAAGTTATCTGGTAATGCTCAAT
TTGAAGGATATAGTGTCGATCTTATTCAAGAAATAGCCCGTATTCTAAAATTCAACTACA
CTATCCGTCTGGTACCCGATGGTCGTTATGGTTCGTATACTAGAGAATTGAAAGAGTGG
GATGGAATGATTAAGGAATTGTTAGATCAAAAAGCAGATCTAGCTATAGCTGATCTAAC
AATCACATATGATCGTGAGCAAGCAGTGGACTTTACAATGCCATTTATGAATTTGGGTAT
AAGTATACTCTACAGAAAGCCGGTAAAACAACCTCCAAATTTATTTTCTTTCTTGAGTC
CATTAAGCTTAGACGTTTGGATTTACATGGCTACTGCATACCTTGGTGTTTCAGTTCTCT
TATTTATTTTAGCAAGATTCAGCCCATATGAATGGGAAAACTCCAACACGATCAATAATC
AAACAACTATGATGGAAAATGAATACACACTTTTAAATTCACTATGGTTTACTATAGGAT
CACTTATGCAACAAGGATCTGATATTGCCCCAAGAGCGATATCAACACGGATGGTGGCT
GGCATGTGGTGGTTTTTCACACTAATTATGATATCATCATATACAGCAAATCTTGCAGCT
TTTCTTACAGTAGAACGTATGGACTCTCCAATTGAAAGTGCTGAAGATTTAGCTAAACA
AACTAAAATTAAGTACGGCGCATTAAAAGGAGGAAGCACCGCAGCTTTTTTTCGCGAC
TCCAACTTTTCGACTTATCAACGAATGTGGCATTTCATGGAAACTGCAAAACCTCCCAA
TGAGGTTTTTACAAAAAGTAATGTTGAAGGCGTCGAGCGAGTTGTCAAAGGAAAAGG
AAGTTATGCATTCCTAATGGAATCTACATCTATTGAATATGTCATAGAAAGAAACTGTGA
ATTAACTCAAATTGGAGGACTTTTAGATTCAAAAGGGTATGGAATTGCCATGCCTCCAA
GTAAGATATCCTATAATGACGAAAATTGTTTAATTAATAAAATAAGTACTATGAACTAA

>CcGR24[ORF 1161 bp] Accession Numbers: MN616787

ATGGCGCTCCTCTTCGCCGTCATCGTCATTATGGTCACCACTGCCATGTCTCTGCCGCCG
GTCATTAGGATTGGTGCAATTTTTACCGAAGACCAGAAGGACAGCCCCTCGGAGTTGG
CGTTCAAGTATGCCGTCTATAAGATTAACAAGGAAAAGGTCCTCTTGCCCAATACCACG
ATGGTCTACGATATCCAGTACGTGCCTAAAGACGACTCGTTTAGAACGTCAAAAAAAG
CTTGCAAGCAGATGTCGCGCTCGGTGCAAGGACTTTTCGGGCCAGCAGATCCATTACT
AGGAGCCCATGTGCAAAGTATATGCGAAGCCTTGGACGTGCCACACTTGGAAGCACGA
GTAGACTTTGAGCCAAGTTTCAAAGAATTCAGTATCAATCTTTATCCGGCGCAAGATCA
TCTAAATCGAGCGTTTCGTGATCTTATGTCATTCCTCAACTGGACTAAGGTCGCTATCAT
CTACGAGGAAGACTATGGGTTGTTTAAGCTGCAAGATTTAGTGAAAGCGCCACCTTCG
CCGAAAACGGAGATGTATATTCGCCAGGCTGGACCTGGTTCATACAGACAAGTACTTA
GGGAAGTTCGACACAAAGAAATTTACAAACTCATCATTGATACAGATCCCATGTATATG
CAACAATTCTTTAGAGCAATATTGCAACTACAAATGAATGATTATAGATATCACTATATGT
TCACAAGTTTTGACATTGAAACGTTCGATCTCGAAGACTTCAAGTACAATAGCGTAAA
CATGACTGCCTTCCGATTAGTTGACCTGGACGAGCCAACGGTTGCGGATACGCTCAAG
CACATGGAACGTTTCCAACCGATCGGCCATACGATTCTCAACAGATCGGGAATCATACA
GGCGGAACCGGCATTGGTGTACGATAGCGTGCAGGTATTGCGCATGGATTGGCAGCGC
TGGACCGCAGTCACGTGCTTAGACCTGCCAATCTGTCGTGCGATAGGGAGGAACCATG
GGACGATGGACTGTCGTTGTACAACTATATCAATTCGGCGAGTACAGATATCTTTATTGA
GTGTTGGATGTAACATTTGTTTTTCAACAACGTTACGTTTAAAAATATTTTCTACCATTTT
CAACTTAAGTTCTTTAATGACACGTAAAATTACATTTTTTTAA

>CcGR25 [ORF 246 bp] Accession Numbers: MN616788

ATGCATTGCCGACATCACTCGCACTCATCCCACACTCTCGCTCATTCGCACAAACGCCA
GATAGCGCCGACGAAGCTGAGACGAGTCTCTTCAGGCATGGCCTTGGCTCAAGGAGG
TGACCATTCTGAAAGCTTAATGTGGAGGTTCGACTGCGAGCTTGCGGGTGAGCCAGAT
GTGATAATATCGCCGGCGCCACCACCACCACCACCACCACCGCCAATAGCGCGCA
CTACGACCCTTTGA

>CcGR26 [ORF 1596 bp] Accession Numbers: MN616789

ATGAATCCTTTGGCTGTGGAGATTTGGTTATACGTACTAGCTGCCTATATGCTTGTCAGC
TTTACCCTCTTCGTCATGGCAAGATTTTCTCCGTACGAATGGAACAACCCACATCCATG
CCTCGGCGAGACGAATGTCGTGGAAAATCAATTTACCATCAGCAACAGCTTCTGGTTC
ATCACGGGTACTTTTCTGAGGCAAGGCAGCGGATTGAATCCCAAGGCAACGAGTACGA
GAATTGTCGGTGGTATATGGTGGTTCTTCACTCTCATCATCATCTCGTCGTACACGGCTA
ATCTCGCTGCTTTTCTCACCGTTGAGCGTATGATTACGCCCATCGAAAATGCCGCTGATC
TTGCCGACCAGACTGATATACCTTACGGTACACTGGAGGGTGGTAGTACTATGACATTT
TTTAGGGATTCGAAAATAGCAATATATCAGAAAATGTGGAGGTTTATGGAGTCAAAGCA
ACCTTCCGTATTTGTATCCTCGTATGAGGAAGGCATCAAACGAGTACTCGAGGGCAATT
ATGCGTTTTTGATGGAATCTACGATGTTGGATTATGCAGTGCAGAGAGATTGCAATCTC
ACGCAGATTGGCGGCCTATTGGATAGCAAGGGATATGGTATTGCAACGCCCAAGGGTTC
ACCGTGGCGCGATAAAATATCCTTGGCGATTCTGGAGCTGCAGGAAAAGGGCGTGATT
CAGATTCTTTATGATAAGTGGTGGAAGAATACAGGAGATGTGTGCAATCGAGATGAGA
AAAGTAAGGAAAGTAAAGCGAATGCTCTCGGCGTCGAAAACATTGGTGGAGTATTCGT
GGTACTATTGTGTGGCTTGGCCTTAGCGATCCTCGTAGCTATCCTCGAATTCTGTTGGAA

CTCAAAGAAGAACGCCCAATCGGATCGGTCGCTGTGCGCGGAGATGGCTTCGGAGTT
GCGTTTCGCAATTCAATGTGGCTCGCGTCAACGCAAGCTTCGAAATGCAAGCTCGAGT
TCTTCGGCTGGTGCAATTGCCTGTAGCCGTTGCAGTTCGCGCTCGTCAATGAGACGAA
GCGTCAGATACAGGGATCCATCACAGCGTCGCGAGACAAGCCGCGAAAGACGCGCAA
GGGAATTACTTCAACATCAACAACAACATCATCAATCGCACGAAGAGACGACTTATGT
GCCAAGCATCGACATACCTTGGTTAAATGCAGGAGGCTGGTGCGGTGGCGATGACGGA
GATGAAGAAATCATTGAGTTACGAGCGAGTGACACTAGACGAGTGCAGCTTGGGGAG
CGGAGTATTGGGTGTAGTAGACGGGAGCATCACCTAGCATCGGTCACAGACGACTTGC
CAACAACAACAGCAGCATCAGCATCAACAACAACAGCAGCCGTATCAGCAACAACCG
AGCAGCAGCAGTACCAGCAGCAGCACCAGCAGCACCAGCAATTGCAGCAACGCGCAC
ATTCGATCCCAGCATCACATGCATTGCCGACATCACTCGCACTCATCCCACACTCTCGC
TCATTCGCACAAACGCCAGATAGCGCCGACGAAGCTGAGACGAGTCTCTTCAGGCATG
GCCTTGGCTCAAGGAGGTGA

>CcGR27 [ORF 1764 bp] Accession Numbers: MN616790

ATGGCCTATTTGAATTGGACACGTGTGGCCATTGTCTACGAAGACGATTACGGTCTAGT
AAAGCTTCGGGAGTTGGTGAGATCACGAAGATCGCAGGACATGGAAATCTATCTGCGA
CAAGCAGATCTCGACTCTTATAGGCAGGTCCTGAGCGAGATCAAAGCCAAGGAAATAC
GCAATCTTATCGTCGACACCAAACCCGGAAATATGCATCATTTTTTGCGGATGATACTTC
AGCTACAGATGAACGACTACAACTATCACTACCTCTTCACTACTTTTGATATCGAAACG
TTCGATCTCGAGGACTTCAAGTACAACTTCGTCAACATTACTGCCTTTCGACTGGTGGA
CGCTGATGATGCGGAAGTACGTGGCGTCCTTAGGGACATGGAGAAGTTTCAGTTTCAA
GGCAACAACTTACTCAACAAATCACGTGTTATACAGGCTGAGGCAGCACTGATGTACG
ATAGCGTGCAGGTGTTCGCGGTAGGACTACGTACCTTGGAACAGTCGCATGCACTAAG
GCCAATGAACATCTCTTGCGAATTGGAACATCCATGGGATGGTGGACTTTCTCTTATCA
ACTACATCAATACGGTGGACAGGAAAGGGATTTCCGGGCCCATAGGTTTCAAGGAGGG
TCGCCGGATACAATTCAAGCTTGACTTGCTCAAGCTCAGGCAGCAGTCGTTGGTTAAA
GTGGGTGAATGGAGACCAGGGTCAGGGGTTAACATTACAGACAAGTCGGCATTCTTCG
AAGCCGGAACCACCAATGTCACCCTGGTGGTCATCACCATTCTCGAAACCCCATACGT
GATGCTGAGAAGCAAAGGCAACTTTAGTGGAAACGATCGCTACGAAGGCTTTTGTATC
GACCTGCTCAAGGAAATAGCGCACATGGTCGGCTTCGCCTACAGGATCGAGCTTGTCC
CGGATGGCAAGTACGGTGTCTACGACTACGAGACTGGCGAATGGAATGGTATCGTCAG
GCAACTCATGGACAAGAAAGCAGATCTAGCAGTAGGCTCAATGACGATCAACTACGCA
CGTGAGAGCGTCATAGACTTCACCAAACCATTCATGAACCTTGGTATCTCGATTCTCTT
CAAGGTACCAACAAGTCACCCCGCGCGCCTCTTCTCCTTCATGAATCCATTGGCAATAG
AAATCTGGTTATACGTATTGGCTGCTTACATTCTCGTCTCGGTAACCATGTTCGTAGTGG
CACGTTTTTCACCCTACGAATGGAACAATCCACATCCGTGTCATTCGCAGAATACAGAG
ATCGTGGAAAATCAATTTTCGTTGGCCAATAGCTTCTGGTTCACCATTGGTACTCTAATG
CAGCAGGGTAGCGACCTCAACCCAAAGGCGACAAGCACGAGGATCGTTAGTGGAGTC
TGGTGGTTTTTCACCCTCATCATAATCTCCTCGTACACAGCTAATCTCGCGGCTTTTCTC
ACCGTCGAGCGCATGATAACGCCCATTGAGAATGCCGAGGATCTTGCGAGCCAGACTG
ACATCTCTTATGGGACACTCGAGAGCGGGAGTACTATGACTTTTTTTAGGGATTCAATG
ATCGAGACGTACAAGAAGATGTGGCGATTCATGGAAAATAAAAACCTTCTGTATTCGTT
CCTACTTATGAAGAGGGCATTAAGCGAGTTCTTCAAGGGAATTATGCCTTCCTCATGGA

ATCAACGATGCTCGACTATATCGTTCAGAGAGACTGCAATCTTACACAAATTGGTGGCC
TATTAG

>CcGR28 [ORF 1353 bp] Accession Numbers: MN616791

ATGTACGATAGCGTGCAGGTGTTCGCGGTAGGACTACGTACCTTGGAACAGTCGCATG
CACTAAGGCCAATGAACATCTCTTGCGAATTGGAACATCCATGGGATGGTGGACTTTCT
CTTATCAACTACATCAATACGGTGGACAGGAAAGGGATTTCCGGGCCCATAGGTTTCAA
GGAGGGTCGCCGGATACAATTCAAGCTTGACTTGCTCAAGCTCAGGCAGCAGTCGTTG
GTTAAAGTGGGTGAATGGAGACCAGGGTCAGGGGTTAACATTACAGACAAGTCGGCAT
TCTTCGAAGCCGGAACCACCAATGTCACCCTGGTGGTCATCACCATTCTCGAAACCCC
ATACGTGATGCTGAGAAGCAAAGGCAACTTTAGTGGAAACGATCGCTACGAAGGCTTT
TGTATCGACCTGCTCAAGGAAATAGCGCACATGGTCGGCTTCGCCTACAGGATCGAGC
TTGTCCCGGATGGCAAGTACGGTGTCTACGACTACGAGACTGGCGAATGGAATGGTAT
CGTCAGGCAACTCATGGACAAGAAAGCAGATCTAGCAGTAGGCTCAATGACGATCAAC
TACGCACGTGAGAGCGTCATAGACTTCACCAAACCATTCATGAACCTTGGTATCTCGAT
TCTCTTCAAGGTACCAACAAGTCACCCCGCGCGCCTCTTCTCCTTCATGAATCCATTGG
CAATAGAAATCTGGTTATACGTATTGGCTGCTTACATTCTCGTCTCGGTAACCATGTTCG
TAGTGGCACGTTTTTCACCCTACGAATGGAACAATCCACATCCGTGTCATTCGCAGAAT
ACAGAGATCGTGGAAAATCAATTTTCGTTGGCCAATAGCTTCTGGTTCACCATTGGTAC
TCTAATGCAGCAGGGTAGCGACCTCAACCCAAAGGCGACAAGCACGAGGATCGTTAG
TGGAGTCTGGTGGTTTTTCACCCTCATCATAATCTCCTCGTACACAGCTAATCTCGCGGC
TTTTCTCACCGTCGAGCGCATGATAACGCCCATTGAGAATGCCGAGGATCTTGCGAGCC
AGACTGACATCTCTTATGGGACACTCGAGAGCGGGAGTACTATGACTTTTTTTAGGGAT
TCAATGATCGAGACGTACAAGAAGATGTGGCGATTCATGGAAAATAAAAAACCTTCTG
TATTCGTTCCTACTTATGAAGAGGGCATTAAGCGAGTTCTTCAAGGGAATTATGCCTTCC
TCATGGAATCAACGATGCTCGACTATATCGTTCAGAGAGACTGCAATCTTACACAAATT
GGTGGCCTATTAGATAGTAAAGGTTACGGTATAGCTACGCCCATGGGTGAGTCTAAAGA
ATGA

>CcGR29 [ORF 1743bp] Accession Numbers: MN616792

ATGGAGAAGTTTCAGTTTCAAGGCAACAACTTACTCAACAAATCACGTGTTATACAGG
CTGAGGCAGCACTGATGTACGATAGCGTGCAGGTGTTCGCGGTAGGACTACGTACCTT
GGAACAGTCGCATGCACTAAGGCCAATGAACATCTCTTGCGAATTGGAACATCCATGG
GATGGTGGACTTTCTCTTATCAACTACATCAATACGGTGGACAGGAAAGGGATTTCCGG
GCCCATAGGTTTCAAGGAGGGTCGCCGGATACAATTCAAGCTTGACTTGCTCAAGCTC
AGGCAGCAGTCGTTGGTTAAAGTGGGTGAATGGAGACCAGGGTCAGGGGTTAACATT
ACAGACAAGTCGGCATTCTTCGAAGCCGGAACCACCAATGTCACCCTGGTGGTCATCA
CCATTCTCGAAACCCCATACGTGATGCTGAGAAGCAAAGGCAACTTTAGTGGAAACGA
TCGCTACGAAGGCTTTTGTATCGACCTGCTCAAGGAAATAGCGCACATGGTCGGCTTCG
CCTACAGGATCGAGCTTGTCCCGGATGGCAAGTACGGTGTCTACGACTACGAGACTGG
CGAATGGAATGGTATCGTCAGGCAACTCATGGACAAGAAAGCAGATCTAGCAGTAGGC
TCAATGACGATCAACTACGCACGTGAGAGCGTCATAGACTTCACCAAACCATTCATGA
ACCTTGGTATCTCGATTCTCTTCAAGGTACCAACAAGTCACCCCGCGCGCCTCTTCTCC
TTCATGAATCCATTGGCAATAGAAATCTGGTTATACGTATTGGCTGCTTACATTCTCGTCT

CGGTAACCATGTTCGTAGTGGCACGTTTTTCACCCTACGAATGGAACAATCCACATCCG
TGTCATTCGCAGAATACAGAGATCGTGGAAAATCAATTTTCGTTGGCCAATAGCTTCTG
GTTCACCATTGGTACTCTAATGCAGCAGGGTAGCGACCTCAACCCAAAGGCGACAAGC
ACGAGGATCGTTAGTGGAGTCTGGTGGTTTTTCACCCTCATCATAATCTCCTCGTACAC
AGCTAATCTCGCGGCTTTTCTCACCGTCGAGCGCATGATAACGCCCATTGAGAATGCCG
AGGATCTTGCGAGCCAGACTGACATCTCTTATGGGACACTCGAGAGCGGGAGTACTAT
GACTTTTTTTAGGGATTCAATGATCGAGACGTACAAGAAGATGTGGCGATTCATGGAAA
ATAAAAAACCTTCTGTATTCGTTCCTACTTATGAAGAGGGCATTAAGCGAGTTCTTCAA
GGGAATTATGCCTTCCTCATGGAATCAACGATGCTCGACTATATCGTTCAGAGAGACTG
CAATCTTACACAAATTGGTGGCCTATTAGATAGTAAAGGTTACGGTATAGCTACGCCCAT
GGGCTCGCCTTGGAGAGACAAAATTTCGCTGGCAATTCTAGAGCTGCAAGAAAAAGG
CGAGATTCAAATTTTATACGACAAATGGTGGAAAAGTCCAAGTGACACTTGTATGAGA
AATGACAAAGACAAAGGAAGCAAGGCTAATGCTTTGGGTGTCGACAATATAGGCGGGA
TTTTCGTGGTATTGTTGTGCGGATTAACATTCGCTGTGCTCATTGCCATTTTCGAATTCT
GTTACAATTCCAAAAGAAATGTACCTGTTGAGCGCCGCATGATGATTTCGACGTCGAAC
ATAAAAACTGTTCAGGCATTAAATTCAAGTATTATTTGTCATTGA

>CcGR30 [ORF 975 bp] Accession Numbers: MN616793

ATGGGAGAATTGAAAAGATCAACTATAATATTCAAAAGTTTGTATTATTATGAAAAAATG
CTTGGACTTTGCCCATTTATCATTCACAAGGATACGCTGAAAATGTCATATTTGGGAATA
ATCTATAATCTCGCAATATCGGTCTTATACAGTATTTGCTTTATATTGATCATTGAGTGTCG
GTTCGAACTTCGTTTACCAAGAGAAACCACGTTAACGATCACAATGGATGCTTTGGGC
TTGCTTTTTCAATATTGTACAGTAGTTTGTTCTTGGTTAACTGTCATTTTTCAATATAATC
GGTTGAAAAGTATATTTAACGATTTCAAAAAAGTGGAAGAATTAGCCAAAACCTTGAA
CATAAACGTTTATGAAAATCGATTGAAAAAACTACGAAATATTTCAATGCGTTTATTGAT
TATCAATGCATTCTATTTAGCTGTATTTTTAGCAGATCACTACACGCTGTCTCTCTATAAA
AAATTTCAAGAACAAGCAAGTGCCTGGATTTGGTACAATGTACCGAAAATAGTACTCTA
CAATGTTGGTGCTATCTTCTTGGAGTTGATGCTTGTATTAAAACACAATTTCAGAGCTCT
GAATAAGTTACTGAGATATTATTTTTCAACAAAAACTGATGAATTTCTATTTCGAAATTT
CTCAACTACTTCAAAGGCGTTTATAAAACTTCACAAGATCGGACAGTTCCATGAAAGC
TTATCGGACCTTCTAGAACATATTACTAGTTTTTTCAGTCTTTCGATTTTGTTTGCGATCA
TAGCAACTTTTATTCACTCTTTTCTAGACATTTATGCCGTCTATCAATATTTGACTTATAG
ACGAATGTGCGAAGTAGGCGATTTCAGCATATATTCACTCAATATCATATGGATGATGAC
GAAATTTCTGACTTTATATTTTATATGTGGAGTTCCGGATTCGGTTTGTACAGAGGCATG
TGCTTTGACAGCTTGA

>CcGR31 [ORF 1731 bp] Accession Numbers: MN616794

ATGAGAAAGCAAGATTTGGAAAAAAAAACGGGATTGATGACGTTTGACAAAACTAAG
CAGCGACAATTCCAACTTAAAGTGATGAATATTCAAGCGGGAAGTGAAACTACTGTAG
GAGAATGGAATGGTAAAGAATTAGTTATTAACAGAAATGAAAAAGAAATAAATAATAC
GCTAACTGAAGCTGCCCAAAATAAAGTCTTTAAAGTAACAACGAGGATTGGACCTCCG
TATGTTATGGCAGCTGAAGGTGATACGAAGGGTAGACAAATTGGAGATAAAATGTATCA
AGGCTACTGTATAGATTTAATTAGTAAAATCGAAGAATTCTTGAAAATTAAATGTGAATT
TGAATTGGTTCCTGATAAACAATACGGAGCATTAAATCCACAAACCCATCAATGGAACG

GACTTGTAAAACAACTTCTAGAACTTAAAGCTGACTTTGCAATATGCGACTTGACAATC
ACATCAGAAAGACAAAGTGCTGTGGATTTTACAGCTCCATTTATGAATCTTGGGATTAG
TATTTTGTTCAGTAAACCATCAAAAGTGCCACCAAAATTATTTGCTTTTATGGATCCTCT
TTCTACAGAAGTATGGATGTACATGGCAACAGCTTATTTGATAGTATCACTTATGTTGTT
TTTTCAAGCAAGGATAGCACCGGGCGAATGGGTCAATCCACACCCATGCAATCCTAAC
CCAGATGAATTAGAAAACAATTTTACTCTAATGAATTCTATGTGGTTGACTATGGGCTCA
CTCATGCAACAAGGCAGCGATATTTTGCCAAGAACTCCATCTATTCGTATGGTCGCCGG
CATTTGGTGGTTTTTCGTACTTATAATGGTATCAAGTTATACAGCTAATTTAGCCGCATTT
TTAACAGCAGTAAAGATGGAAGATTCCATAAATGATGTTGAAGATTTAGCAAAACAGA
CCAAAATATCATATGGAGCTGTGAAAGGTGGAGCTACCTATTCTTTTTTCAAAAATTCA
AATACATCACTGTATCAGCGTATATTCAATAGTATGACGGATACGAAACCAACTGTGTTC
ACAAGTACTAATGAAGAAGGTGTTGATCGTGTTATAAAAGGCAAGAGAAAATACGCCT
TTTTTATGGAATCAACTACCATAGAGTATCAAGTAGAACGGCACTGCGAATTACAGATG
GTTGGAACATTACTTGATAATAAAGGATATGGAATAGCTATGCCACCAAATTCACCTTAT
CGTACTATGATTAGTACGGCTATATTACATTTACAAGAAAAAGGAGATTTACAGCAACTA
AAACAGAAATGGTGGAAAGATATGGGCGGCGGAAAATGTTCTGATGATTCTGATGAAC
CAACTAACTCGAATGAATTAGGGATGCCACATGTAGGAGGAGTCTTTCTTGTATTGATG
TTGGGTTGTATTATATCAACACTGATTGCAGTTATAGAGTTTCTATGGAACATAAGAAAA
GTTGCTGTTGAAGAAAAGATTACTTTATTTGAGGCTTTCATCAAGGAAATAAAGTTTGT
CATAAATATATGGGCTGTTACAAAACCTGTTAAGGTTGAGAGGTCTAACAAATCAGCTT
CAATACAAAATCGGAGTTCTAGCAAATCTGATTCAATAAATATTTCTTCTACAAGCTCAT
TGAATAGAGTAAATTTAAGAGATACAATTAATATAGACAAAATGAATACACATGAAGAA
GTTTTAAATGTACATTAG

>CcCSP1 [ORF 378 bp] Accession Numbers: MN616756

ATGACTCCTAAACGATCCTTGATCGTTGTGGCCACGGCCCTCTTGGTACTCGTTGCCGG
TGTCGTTAGAGCCGAGGACAAAAAATATGATTCGAAATACGACAATCTTGACGTCGAG
GCGATCCTTCAAAACGATGCGGAACGTAACATTTATTACGCTTGCTTCATGGATACTGG
ACCATGCCCCAATGAGGCTGCCATCTTTTTCAAGGGCCACGCACCAGAGGCTGTAGTT
ACGTCTTGCAGATATTGCACTCAAAAACAATTAGAGATGTTTGAAAAAATAGTAAGTTG
GTTTGTCGATAATAGTCCTCAGGAATGGAACGCATTAATTGAGAAGACTATCAATGATG
CCAGAAAACAAGGACTCAGCTTTTAA

>CcCSP2 [ORF 354 bp] Accession Numbers: MN616757

ATGAAAGTGATACTTTTCGTGTTTTTGGCCTTTTACGCCGTCGCTGCTGAACAGCTATAT
TCAGACCAATATGATTACGTCGACGTTAGTAAAATTTTGTCAGACGACGCACTCAGAGA
AGAATATTATAACTGTTATATGGGAACGTCACCTTGTCTGACGGCGGACGCTCAGTACT
TCAAAGAAATTCTGCCAGAGGCAGCTTTAACTAAATGTGTAAAATGTACCGACAAGCA
GAAAGATAATTTCCAAAAAATAGCTACTTGGTTTACAAAAAATCAGCCAGAGAAATGG
GATGCTTACACGAAGAAAGCAGTCGAAATTTACAATCAATCCCAAAAAGCGCCGGAAT
AG

>CcCSP4 [ORF 1605 bp] Accession Numbers: MN616759

ATGGTGACACGACCTCAATTTGTACAACCTCCTGCTCAAGGTAACGTCGGTGTGTCCTT
TCACGAAAAGACATGGCTAGAACAATACGCCTCGGCCAAGGTGCCCAACAGCATTGA
GATGCAGGCAAGGTTCCAACAAAAGCAACTACAGGAGAAGGAGCAAAAACTCCTACA
GTTGTACGATCAGCAGCAGCAACGAGCGCATCAAGTCGCACAAAGAGGTAGCGCCGG
CTCTAATGGCTCCAATCACAGTATCACCGCTAACGCCAATAAAGTGAAACAATTATTCG
CCGAAGAGAGACGACAACAGAACGGTGTTAAAGGTATCGACAAGAGTTATCCTCTCG
AACCTTTGAAAAGCAAAAAAACATCGCCAATCAATAAAACAACCAACGCCAAATCAA
CTTCCACCAGTACCATCATCGCCAATATCAATAATAACAATAACAACATGATAAATCGAA
AGTCAAATTCAAACGTCGCTACGAAATGTGTGATAAAGGCAGACGCGAACAGCAACGT
AAGTAACAGCCGAAGTATCAACGTACAGCAACAGCAGCACGAGAGAGAGAACAACTT
TGCCTCGAGCAGTCGACAAGAGAACTTGTCTCGCGAATCTTACGGGAACTCTATTCGC
TATAACGAAGCGGCCAACAGCAGCGAATCGATTAGGCAGGTCAACGGACACACTGCA
ACGAAGAACGGTTATCACTACGAGATAAACATCGATGAGGTGATCGATAACGAAGCAC
TGCAGCGCAACCGCATGCTAGCGAAATTTCAACCAGGCGACATCGAGAGGAGACGTC
GTCAATTGAGTGCCGACATCATCGACGACGACGACGACAACGACGACGACGACGACG
ACGACGACGACGACGACGACGACGACGACGAAGACGACCACGGTAGTGAATTCCCTA
TGGATACACGGTACGATGTTAGCACCTCGCGGAAATCCTCCCTCAAAAATACCAAGAG
CAATCCACCGAGCACTACTGCTTCTGCTGTTGCAGCAACCAAGAGAACGATTCCCAGT
AATACGACGAGTTTAAAGAAGGCTGATTCCATTACGAAGATGGTGCAATCCGAAGCTG
TCCAAATGGTAAAGAACGTATCGCCGTATCCTTCGTCCAAAAGTCCCGATAGCAACGA
GATGAAAAATCGATCGATGGTCAAATCAACGAGTTTGAAACTTCTCAGTCGTTTCAATG
TTTCCAACGGTGACGCGACACCTACTGTTCCTACTGAAACCGATCAACCGAGCGTGTC
GCGAAAATCGAGCGTCCATACTGTCGAGCTTTCACAACACGACGAGATCAAAACAGCT
GCACCAGTTGTGAGCAAACCCGATTCTCAGAAATCCAATTGGCGTCGCAAGCACGAG
GACTTTATAAATGCGATTCGTTCGGCGAAACAAATGCAAGCACACTTAGCAGCAGGTG
GTAAACTAAGTGATTTGCCACCACCTCCACCAACCGATACAAGTGACTACGTACAATGT
CCTCATTGCTCGCGTAAATTTAACCAAGGAGCTGCTGAACGACATATTCCCAAGTGCGA
GGCTATGCAACATAATAAACCGAAACCTCGACCCAAACGCTAA

>CcCSP7 [ORF 324 bp] Accession Numbers: MN616762

ATGAAGCACACGTGCGCCGTCGTTGTCCTGATGCTACTGCTGCTGCTTGCAATCGTCGC
TTCTGCTCAAGACGTCAATATCTTACTGCAAAACAAAAATCTCGTTAGTCGAGAAATCG
GTTGTGTGTTACAGCGCAACCCTTGTGACGTCATCGGCAAACAAATTAGAGGTTTATTA
CCAGAAGCTTTGAACAACGGCTGCGGTCGGTGTACTCCTCAGCAAGCGACCAATGCC
AAGAAGCTCATTGCCTACATGAAGAAGAATTATCCAAACGAATGGGTTATGATCGCACA
GATGTATGGCCGAGCAAAAGCTGTTTACTAA

>CcIR75p [ORF 1764bp] Accession Numbers: MN616804

ATGCTTCATAATCATCTGAGTTTCGACGATTTTCAAAAAGATGATTTTCCTTTGCACAAG
ATTGTAATTTTTCTTGATTTCAAGTGCCATGGAGCAAGAGAATTTCTGCTGAAAGCTAAT
AGTTCGGAGATGTTTTCAGCCCCTTATAAGTGGATCATATTTCAAGATCTCGAACATAGC
TCTCCTGACAATTGTACAGATGGATGTGCGTTCAAAGATTTTTATAGTTATGCAATGTAT
CCAGACAGCAGCGTCGTCATTTTACAAAAATTGTCAAAAGAACGTGTACAAATTGTAT
CTATATACCGACCAAGTCCAGTACGAGACATGATCGTTGAGAATCTTGGATATTGGAGT

AGCACTAATGGCACTAAGTGGCATAATTTGAACATTGCATCACAAAGACGAAAGAACC
TTCAAAAAACTCCCTTGAAATCGAGTATTGTGGTCACGAATCCAGATACGTTAAACCAC
TTGACCGATTATCACGACAAGCACGTAGATACTATCACAAAATGCAATTTTGTGTGGCT
CCATCAACTTATCGATGCAATGAACGCAACGGTGACGTACAGTATAGTTAACACCTGGG
GATATCGCGATAAAAATGGTTCTTGGACTGGGATGACTGGCCAACTGAGCCGCAAAGA
GATCGACATTGGAGGAACATCGATGTTCATCATCGGAGACCGATGGAACGACGTACATT
TCATTCCTTTGTCAACACCTACCAGACAAGCGTTCATTTTTCGACAGCCTCCCCTGTCG
TTCGTCAGCAACTTGTTCACTCTACCCTTCCGTCCGTCCGTATGGATAGCGATAGGTATC
CTTCTGATGATAATTTTTGCAATGCTCTTGCTAGCTACGAAATGGGAGTGGAGAAAGGT
ATACGCCGACCGAGAATTCAGCGAGAATGAGCCTAAACCAAATTTGAGCGATCAACTG
TTGTTAATACTAGGCGTTTGCGCTCAACAAGGATTTGGACGCAGTCCGTATACGGTACC
ATCTCGAATAGTTCTCTTGATGCTCCTGTTGGCCGTGTTGAATTTGTACGCTTCGTATTC
GGCCAACATCGTTGCTCTTTTACAATCTACAACGACGTCGATAACGAGCTTGAAAGATC
TACTGGAGAGCCCTATAAAATGCGGAGCCAACGATATCGTTTACAACCGTCATTATTTC
AAACTGGAAAAAGATCCGGTGAAAAGAGCTATTATCGACAAGAAAATCGAGCCTAAA
GGCAGTAAAGCTAATTGGATGACTGCCGATGAAGGAATCAGTCGAGTGCGACAGGGCT
TCTTTGCTTTCTTAATAGAAACCGGTCCTGGGTACAGGATACTTCAGGAAACCTTCGAA
GAGGATGAAAAGTGTGGATTTCGGGAAATGTATTTTATCGATCACTTTGATCCAATGTTT
GCCATCGTCAAACGTTCGCCTTACAAAGAGCTGATCAGAGTCAATTCCTTAAAAATTTG
GGAATCAGGATTAAAATCCAAAGAAATGTCACGTTTGTATACGAAAAGGCCCCCTTGC
AATGGCCGCAACAAGTTTGTTAGCGTTGGCTTGAACGAATGTTATTTTGCATTCTACAT
CATTGGTTACGGAGTACTATTCGCTATTTTAGCGTTCCTCGTCGAAATTCTATCGAAGAA
AAGTGGTTCTCTAAGAAAACGTCAACCCGTTGAGTCGACTGCACGGACAAGTTTTGCA
CAACGCAATTTGCAACAAAACAGTGCGCGCGAATCACCGTTCTCCGCTTCGTAA

>CcIR64a [ORF 1356bp] Accession Numbers: MN616798

ATGATAAAGCAAAGCGTCTATATGCATACATCTGGTTTGCAACGTGTATTACTAAATATT
AATCAAGTAATTACGATAATTATAATAATTCGACAATTAGTACCAATTGCTAGCGAAATA
GTAGAACCAGAATTTACTTTAAATTATTTTGCTGAAAAAAAAGTGCGCCAAATTACCGT
ATTCGCTTGTTGGAATAATACAGATATTTTGAGATATACTAGGCACATTATGAAAAATAC
TCAAATATCCTATAACGCTATTCAAGATAATTTGACCATTAGAAATATTTTAAAATTTAAT
TATTATCGAATTGGCGTTGTTCTTGATATTGATTGTTCAGGAAGTCATTCTATATTGAATC
AGTTTTCAGCTGCTCGTTTATTATACAATGAATCATACGTATGGCTTGCTGTTACTACATT
GCTAGAAGTACCAACGATAATTTTAAGTAAACTTCCCCTTAGAGTTGATACCGAATTCG
TAGTAGCTTTGGCACATAGTGATAGATACATCCTTTACGACGTCTACAACCATTCGTATC
GACATGGCGGAAAACTCAATGTCACTGCTATGGGTTTTTGGAATAAACAGAGTGGATT
GAAAAATTATTTGACACAATACAAATACAAGAGATGTCAAAATTTACATGGAATGATGC
TCAATTTTTCTACCATTCTGACGAATAAACCTGACTCTGATATGGAAACATACCTTACAA
TTCCAAAAAATCCTCATCTGGACACATTGGCACGTTATCATTATGGCTTAGTACTCTATC
TTCGTGATATATATAATTTCACGATCAATTTGAAATTAGATACTGTAAGAGGTTACCGTAG
ACCAAACGGAAGTTCCGATGGTATTGTCGGTGACATGCTTAAGGGTACTGTAGATGCG
AGTGCTTGCTACTTTGAACAGAGAGTAGAAATACTAGATTCCGTTGAATATACTGTGCC
TACATACGAATTGAAACGTCTCATGTTTTTTCGACACCCAACAAAAGCTGCTATGAGAA
ATCAATTTCTAATGCCATTAGCTGAAGATGTCTGGTGGTTAGCTTTAAACTTCAGCGTAG

TTTACTGGATTTTTTTACTTATTTCTACAAAAGTTGAAGATTATTTTCGAACTAATGGAG
ATCGATTGGTGCATCCGGATGCACCGTCAGATACGATAATTGAAATTATTGCAGCAATAT
CGCAACAAGGATCAAGCATTGAACCTCGTTTATTTTCTGGGCGAATAGCATTCCTTACT
CTTTTTGTATGGTCACTTGTAATGTTTCAGTTCTATTCCGCAAGTATAGTCGGTTCATTGC
TAGCTCCACCAAAAAGATTATTAATACACTTTGGGATCTAG

>CcSNMP2 [ORF 1563bp] Accession Numbers: MN616916

ATGGCGATTAATCGTATGATGAAATTTTCCATCGCGGGCTCATGTTTGTTAATGTTTGGA
ATTCTGTTTGGGTTTATGACGTTTCCCAAGCTTCTGAAGGGTGGCATTCATAAAATGGT
CAATTTGAAACCTGGGACCGACGTGCGAGCATTGTGGAGCAAAATTCCATTTGAAATC
GATTTTAAAATTTACTTATTTAACGTAACAAATCCAGATGAAATAAAGAGTGGCGCAAA
ACCAATAGTCCGCGAAGTTGGGCCATACTTTTTTGAAGAGTGGCACGAAAAGAACGAT
CTAATCGACGACGATGAAGAGGATACGGTTACTTATTCTCCGAAAAATACATTTATTTTC
AATCCAAAGAAAAGTAATGGACTGACCGGTGAGGAGGAGCTCATGATTCCACACATTT
TTATTTTAGCCATGATATTTGCCACTTTGAGAGAAAAGCCTTCAGCGATACCGTTAATTA
ACAAAGCAATCAACAGTATCTTCAAGAGTCCGGAAAATGTATTCGTCAAAGCGAAGGC
CATGGATTTAATGTTTCGAGGTTTGCCGATTGATTGCTCTGTAACCGACACTGCAGGCT
CGGCTGTTTGTAGCCTGTTAAAGGCCAACACGGACGATCTCATCGTAGACGATCCCGAT
CATTTTCGGTTTGCACTTCTTGGAGCGAAAAACGGCACGACGAGCAAAAATCGCATCA
AAGTTTTACGAGGAGTTAAGCATCAAAATGACATTGGGGTCGTCACTGAACTCAATGG
CAAGAAGAAAATGAGCGTATGGAACGACAGCAAGTGCGATTCTTACGAAGGTACCGAT
GGCTACGTGTTTCACCCTTACCTCTACGCCGAGGAAGATATCGTTTCGTTCGCTCCCGA
CTTGTGTCGCAGTATAGCCGCTTATACCGAATCTACCTTCAAGAAAAATGGACTGCTCG
TAAATCGGTACACCGCCTGGCTGGGAGATCCTGTCAAACATCCCGAGCAAAAGTGTTA
TTGTCCGACCTCGGGCTGCCTCAAAGCGGGCATGATGGATCTTCACAAGTGCGTTGGC
GTGCCGCTTGTAGCCTCGCATCCACATTTCTTTCGAGCAGACGAAGAATACCTGAAAA
CGGTCGATGGCCTAAGTCCCAATGCGGACAAACACATGATATTTATCGATTTCGAACCG
TTCTCCGGGACACCCTTGGAAGCGCAAAAACGATTGCAATTCAATATATGGATGCACA
AAGTTGAAAAAGTTAAAATAATGAAGAATTTCCCGGAGGCCATGTTACCCCTATTTTGG
GTTGAAGAAGGCCTCGTTATACCGGACAGCTTTGTAAAGCAAGTAAAAATGCTTCATAT
GGTCGTGAAAGTGATGAAGTGGCTAACGTGGATAAAAATATTAGCTGGATTGGGAATG
ATTGGTTATGCCGGATTTTTGTACTATCAATCGACGCAGGGAAGCAAAAAGGTTGAAAT
AACAAAGCCTCCAAAATTTGACAATGGATATAAATCGCAAGTATCCACACTCGACGCC
AACAATCTACGGGCTCAAATTCCGCCGTCCATTGATTGA
