# Supplementary material for: Full-Length Transcriptome Survey and Expression Analysis of Parasitoid Wasp Chouioia cunea upon Exposure to 1-Dodecene
Source: Sci Rep. 2019 Dec 3;9:18167. doi: 10.1038/s41598-019-54710-0 (PMC6890788; doi:10.1038/s41598-019-54710-0)
Supplement: Supplementary file 3 — Dataset 4 [file 41598_2019_54710_MOESM3_ESM.pdf]

Full-Length Transcriptome Survey and Expression Analysis of Parasitoid Wasp *Chouioia cunea* upon Exposure to 1-Dodecene

Lina Pan[1], MeiqiGuo[1], Xin Jin[1], Zeyang Sun[1], Hao Jiang[2], Jiayi Han[1], Yonghui Wang[1], Chuncai Yan[1], Min Li[1]*

[1]Tianjin Key Laboratory of Animal and Plant Resistance, Tianjin Normal University, Tianjin 300387, China.

[2]South China University of Technology, 381 Tianhe Road, Guangzhou 510641, China.

***Corresponding author:** Min Li, Tianjin Key Laboratory of Animal and Plant Resistance, Tianjin Normal University, Tianjin 300387, China. E-mail: skylimin@ tjnu.edu.cn. Tel: +86 022 23766673

Table. S3 The integrity of the RNA samples for PacBio sequencing.

Sample name	RIN	28S/18S	baseline	OD260/280	OD260/230
1-Dodecene treatment-1	7.10	0.05	nomal	2.13	1.90
Control-1	7.30	0.05	nomal	2.11	1.89
1-Dodecene treatment-2	7.10	0.02	nomal	2.07	1.82
Control-2	6.80	0.05	nomal	2.11	2.03
1-Dodecene treatment-3	7.40	0.1	nomal	2.11	2.00
Control-3	6.90	0.02	nomal	2.13	1.92

There is a thermal denaturation process of RNA before detection with Agilent 2100 device, which would make 28S of arthropods split into two fragments. Therefore, no 28S/18S could be considered as a determination index for arthropods.
