# Supplementary material for: Full-Length Transcriptome Survey and Expression Analysis of Parasitoid Wasp Chouioia cunea upon Exposure to 1-Dodecene
Source: Sci Rep. 2019 Dec 3;9:18167. doi: 10.1038/s41598-019-54710-0 (PMC6890788; doi:10.1038/s41598-019-54710-0)
Supplement: Supplementary file 8 — Dataset 9 [file 41598_2019_54710_MOESM8_ESM.pdf]

Full-Length Transcriptome Survey and Expression Analysis of Parasitoid Wasp *Chouioia cunea* upon Exposure to 1-Dodecene

Lina Pan[1], MeiqiGuo[1], Xin Jin[1], Zeyang Sun[1], Hao Jiang[2], Jiayi Han[1], Yonghui Wang[1], Chuncai Yan[1], Min Li[1]*

[1]Tianjin Key Laboratory of Animal and Plant Resistance, Tianjin Normal University, Tianjin 300387, China.

[2]South China University of Technology, 381 Tianhe Road, Guangzhou 510641, China.

***Corresponding author:** Min Li, Tianjin Key Laboratory of Animal and Plant Resistance, Tianjin Normal University, Tianjin 300387, China. E-mail: skylimin@ tjnu.edu.cn. Tel: +86 022 23766673

S8 Table. Primers used for RT-PCR and RT-qPCR analysis of olfactory genes of the *C. cunea*.

Primer name	Forward primer (5'-3')	Reverse primer (5'-3')
Odorant binding proteins		
OBP6	GGACTTGGTGCTGGTA	TGACTCGCTATCTCGTT
OBP7	CGACGAATCCATACAAA	TCAGCATCCTCCAAGA
OBP14	GATGTTGCCAAGGAATG	AAGAAGCGAGGTAGAGGT
OBP15	TAATGGAATGCTGGATG	TTAGCGGCAGTCTCAC
OBP16	CTTGTTTGCTGCTTGG	TTCAACTTCGCTGTCG
OBP18	GACTGTCCGAAGAGGTTT	GTGACGATGCGTGATTT
OBP19	TTGACTTAGTAGACGGAACT	ATACTCATCGGGCATC
OBP26	GTTTACAAGAAAGCAGGAA	TCAGCCAGGTCGTTATG
OBP27	TGCTGCGTGCGTGAT	CATTCGGTGAATTGTTCT
Odorant receptors		
OR2	CTTACTTTCCCTTCAACAC	CTCTTCAATACCGACAACT
OR19	CAATACGACGAAGAAGG	ACTAGCTGCGTTACTACAC
OR22	GGTGTTGCTGCTTCAGG	TTGTCACGGTCCGACTT
OR45	ATCCTATTCCTACGGTATTT	TCAGCCATCCCTTCC
OR47	CCCTTATTACGGTTTCC	AATCCTTTCCTGGTGC
OR54	CATTATACTCAACAACTTGCCAAAG	TGCTGCCGCTGTTCACC
OR76	GCGATTCAGTGGTTCTTTGC	CAAGCAGGTTGTTGTGGAAGT
Gustatory receptors		
GR1	AGATTCATCGTCAGCGGTGTT	CGGCCATTGATCCGACAC
GR18	CACCCTCATCATAATCTCC	ATGAATCGCCACATCTT
Chemosensory proteins		
CSP1	GCCCTCTTGGTACTCG	ACTACAGCCTCTGGTGC
CSP4	AGGAATGGGCGTTGG	CTCTGCATCTTCGGGAC
CSP10	CTCGTTAGCGGCAAGA	CAAATGGAAGCCCAAGA
Sensory neuron membrane proteins		
SNMP1	CCACCTGGTACATTCTCG	TCGTAGCAGCGGGAT
